# Potential Risk of Dengue and Chikungunya Outbreaks in Northern Italy Based on a Population Model of *Aedes albopictus* (Diptera: Culicidae)

**DOI:** 10.1371/journal.pntd.0004762

**Published:** 2016-06-15

**Authors:** Giorgio Guzzetta, Fabrizio Montarsi, Frédéric Alexandre Baldacchino, Markus Metz, Gioia Capelli, Annapaola Rizzoli, Andrea Pugliese, Roberto Rosà, Piero Poletti, Stefano Merler

**Affiliations:** 1 Fondazione Bruno Kessler, Trento, Italy; 2 Laboratory of Parasitology, Istituto Zooprofilattico Sperimentale delle Venezie, Padova, Italy; 3 Department of Biodiversity and Molecular Ecology, Fondazione Edmund Mach, San Michele all’Adige (TN), Italy; 4 Department of Mathematics, University of Trento, Trento, Italy; 5 Dondena Centre for Research on Social Dynamics and Public Policy, Bocconi University, Milan, Italy; Santa Fe Institute, UNITED STATES

## Abstract

The rapid invasion and spread of *Aedes albopictus* (Skuse, 1894) within new continents and climatic ranges has created favorable conditions for the emergence of tropical arboviral diseases in the invaded areas. We used mosquito abundance data from 2014 collected across ten sites in northern Italy to calibrate a population model for *Aedes albopictus* and estimate the potential of imported human cases of chikungunya or dengue to generate the condition for their autochthonous transmission in the absence of control interventions. The model captured intra-year seasonality and heterogeneity across sites in mosquito abundance, based on local temperature patterns and the estimated site-specific mosquito habitat suitability. A robust negative correlation was found between the latter and local late spring precipitations, indicating a possible washout effect on larval breeding sites. The model predicts a significant risk of chikungunya outbreaks in most sites if a case is imported between the beginning of summer and up to mid-November, with an average outbreak probability between 4.9% and 25%, depending on the site. A lower risk is predicted for dengue, with an average probability between 4.2% and 10.8% for cases imported between mid-July and mid-September. This study shows the importance of an integrated entomological and medical surveillance for the evaluation of arboviral disease risk, which is a precondition for designing cost-effective vector control programs.

## Author Summary

The tropical mosquito *Aedes albopictus* is a vector for several viruses that can be transmitted to human hosts through infectious bites. In the last 20 years, *Aedes albopictus* has rapidly expanded its habitat to temperate climates and it is now widespread in a large part of southern Europe and the United States. Here, it may cause outbreaks of local transmission of exotic diseases, such as chikungunya and dengue, after the arrival of infected patients through international travels. In this study, we estimate *Ae*. *albopictus* abundance in a subalpine region of Italy, by means of a mathematical model calibrated to data from adult mosquito captures throughout 2014; we then proceed to estimate the probability that an outbreak of local transmission of chikungunya and dengue will occur, following the importation from abroad of a single infectious case at different times in the year. These estimates are useful for planning preventive actions, such as programs of vector population control, as well as for increasing the preparedness of public health authorities in responding to an outbreak.

## Introduction

The invasive mosquito species *Aedes (Stegomyia) albopictus* (Skuse, 1894) (syn. *Stegomyia albopicta*) has spread widely in Europe over the last twenty years, especially in the Mediterranean region [1, 2]. *Aedes albopictus* is a highly competent vector for many arboviruses including chikungunya [3, 4], dengue [3, 4] and Zika virus [5]. Therefore, its broad diffusion provides favorable conditions for the potential spread of diseases that have been until now confined to tropical regions [6], and which are continuously imported into temperate regions by infected international travelers. A major outbreak of chikungunya occurred in Italy in 2007, with over 200 cases [7], and sporadic local transmission has been recorded elsewhere in Europe [8, 9]. A small outbreak of dengue (15 cases) took place in Croatia in 2010 [10], followed by a much larger one, mainly transmitted by *Aedes aegypti* L., in the Portuguese island of Madeira in 2012 [11], with over 2000 cases; autochthonous dengue transmission has been repeatedly reported in France as well [12, 13]. In Italy, chikungunya and dengue are included in the mandatory medical surveillance system [14] to apply timely interventions aimed at limiting the impact of imported tropical infections [9]. In addition to medical surveillance, entomological surveillance is crucial to evaluate potential risks and identify optimal preventive and control strategies targeting the vector population. *Aedes albopictus* populations are highly variable across different sites, depending among other things on local temperature patterns, which strongly influence their life cycle [15], and on the amount and quality of breeding sites available for egg hatching and development of immature stages. The amount of breeding sites also depends directly on many complex factors, including precipitation patterns, air humidity, availability of natural or artificial containers, presence of shaded areas, and indirectly on land use (vegetation index, urbanization level). In particular, precipitations fill natural and artificial containers, creating potential habitats for the aquatic stages; on the other hand, excessive rainfalls may flush larval habitats reducing the overall population of adult mosquitoes [16]. Temperature and air humidity influence the evaporation of water, which is detrimental to the productivity of the breeding site [17] and may lead to complete drying out of the container. The suitability of a breeding site for oviposition may depend, among other factors, on: water surface and depth [18] which vary with evaporation and meteoric water input; nutrient concentration [19], depending on the presence, density and type of vegetation surrounding the container; and shading [20] which depends on land use and vegetation. The combination of all these dynamic variables determines a high spatial and temporal heterogeneity of mosquito population densities.

The purpose of this study is to estimate the potential risk of outbreaks of chikungunya and dengue in northern Italy related to the abundance of *Ae*. *albopictus* mosquitoes. To this aim, we estimated vector abundance by fitting a mathematical model for mosquito population dynamics to novel data obtained from entomological surveillance.

## Methods

### Study area and mosquito data

The study was carried out in the provinces of Belluno (46°08’27”N, 12°12’56”E) and Trento (46°04’00”N, 11°07’00”E), northern Italy. This mountainous area covers a large part of the Dolomites and the Southern Alps. More than 70% of the territory lies over 1,000 m a.s.l. and about 55% is covered by coniferous and deciduous forests [21]. The climate is temperate-oceanic with four main areas: sub- Mediterranean (close to Lake Garda with mild winters), subcontinental (the main valleys with more severe winters), continental (the alpine valleys) and alpine (the areas above the tree line) [21]. The human population as of 2014 is around 208,000 in the Belluno Province and 537,000 in the Trento Province with the majority of the population concentrated in the valley floors.

**Fig 1:**
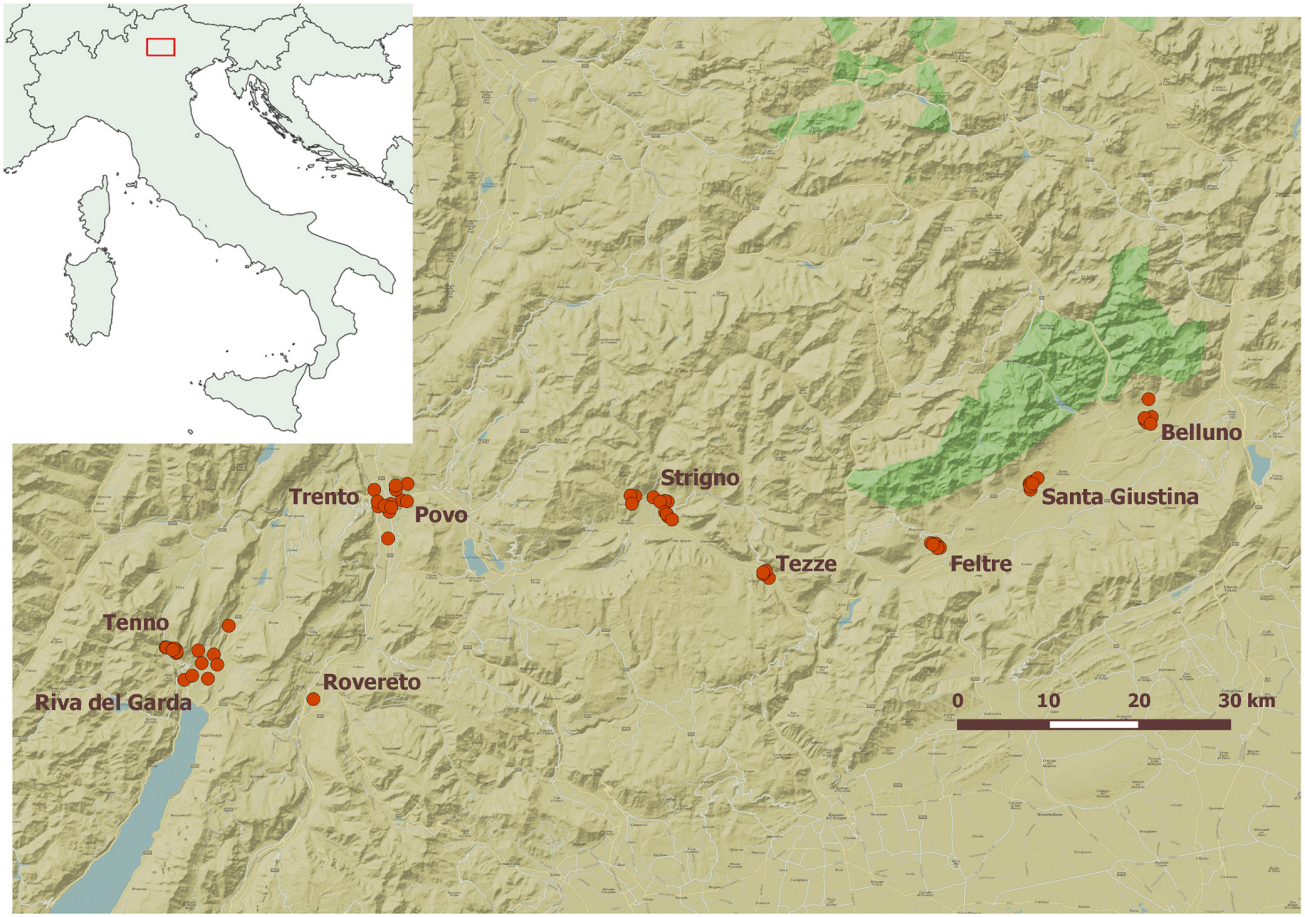
Geographic distribution of trap locations (red circles) and municipalities in the study area.

An entomological surveillance has been initiated in northeastern Italy since the introduction and establishment of *Ae*. *albopictus* in 1991 [22]. Furthermore, *Aedes koreicus* (Edwards, 1917), another invasive mosquito species, has been detected in the Belluno province in 2011 and in the Trento province in 2013 [23, 24]. Therefore, as part of the LExEM project (Laboratory of Excellence for Epidemiology and Modeling, www.lexem.eu), a mosquito monitoring focused on these two invasive *Aedes* species has been carried out in several localities of the provinces of Belluno and Trento ranging from 74m a.s.l. to 650m a.s.l. (see S1 Text). Seventy trapping locations in ten municipalities (Feltre, Povo, Riva del Garda, Santa Giustina, Strigno, Tenno, Tezze, Trento, Belluno and Rovereto) were selected by skilled entomologists in different places such as houses, schools, cemeteries, garden centers, public buildings, farms and tyre storage (Fig 1).

Mosquitoes were collected using Biogents Sentinel traps (Biogents AG, Regensburg, Germany) baited with BG lures and CO_2_ from dry ice. Traps were placed in shaded positions sheltered from wind and rainfall as recommended by the manufacturer. All the collections were conducted in 2014. Each trap was set fortnightly for 24h from mid-April to the beginning of November except the trap of Rovereto which was set monthly from the end of June to the end of September. After each trapping session, mosquitoes were killed by freezing at -20°C and identified using taxonomic keys [25, 26] and confirmed by PCR if found in a location for the first time [26].

### Land surface temperature

The MODIS sensors onboard the sun-synchronous polar-orbiting NASA satellites AQUA and TERRA are designed for global environmental monitoring, providing four records per day with a spatial resolution of 250 m to 1000 m. Most MODIS land products are accompanied by a detailed pixel-wise quality assessment, which facilitates further automated processing such as filtering and enhancement. Land surface temperature (LST) data for 2014 were obtained from the MODIS version 5 LST products MOD11A1 and MYD11A1 with four records per day and a spatial resolution of 1000 m. We applied outlier filtering and gap filling to these data and enhanced the spatial resolution to 250 m [27]. Here we used daily averages calculated from the four records per day of the reconstructed dataset.

### Population model and calibration

We developed a mathematical model representing the dynamics of *Ae*. *albopictus* populations throughout the whole developmental cycle (eggs, larvae, pupae, female adults) within a single mosquito season (April to November). We adopted a compartmental structure similar to other models commonly used for *Ae*. *albopictus* [28–30] and other related species [31]. We used previous estimates of temperature-dependent rates of mortality, development from one stage to the next, and gonotrophic cycle [28] based on experimental data specific for *Ae*. *albopictus* [15]. The model is initialized at April 1^st^ 2014 with a fixed number of 10,000 mosquito eggs. The model robustness with respect to this assumption was verified by means of a sensitivity analysis. Abundance variability across sites was captured by local temperatures as well as by the estimation of a site-specific parameter summarizing the quantity and quality of breeding sites and representing the carrying capacity of the larval population. In other words, larval carrying capacity represents a measure of habitat suitability in our analysis. The process of capture of adults was modeled with a common capture rate across sites that was also estimated during the calibration process. We do not explicitly consider mosquito dispersal, because the average flight range of *Ae*. *albopictus* is much smaller (a few hundreds of meters [32]) than the scale of our risk predictions, which are given at the municipality level. However, the mosquito flight range was implicitly considered in the modeling of the capture process (see S1 Text). The number of captures estimated by the model was calibrated to experimental data by a Monte Carlo Markov Chain approach based on a Poisson likelihood combined over the capture sessions and ten study sites. All details are reported in the S1 Text.

### Association between precipitation and habitat suitability

We investigated the relationship between site-specific habitat suitability (encoded by values of the larval carrying capacity estimated by the population model) and two precipitation variables: the average daily rainfall (*r*_*A*_) and the number of rainy days (*d*_*r*_), calculated for each study site. We sampled 10,000 sets of values for the carrying capacities of the ten sites from their posterior distributions, and computed the precipitations occurring at the corresponding study sites within a given time window. We performed a correlation test with Spearman rank method between each set of carrying capacity values and the two precipitation variables. If the correlation was statistically significant (p<0.05), the value of Spearman’s rank correlation coefficient (ρ) was stored for that test; otherwise we considered the correlation coefficient to be zero. We then computed the 95% credible interval (CI) of the distribution of 10,000 correlation coefficients and considered the correlation to be robust if both boundaries resulted to have the same sign (i.e. either strictly positive or strictly negative). This procedure was iterated over 340 different temporal windows, starting on 34 different dates (between January 1^st^ and August 20^th^ 2014, with interval of one week) and with ten different window lengths (between 4 and 13 weeks). For instance, the first temporal window of 4 weeks starts on January 1^st^ and ends on January 28^th^ while the last temporal window of ten weeks starts on August 20^th^ and ends on November 10^th^.

### Risk of arbovirus outbreaks

We assessed the potential risk of outbreaks of chikungunya and dengue caused by the introduction in the study sites of a single infectious individual, in the absence of control interventions. To this aim, we used mathematical formulae previously derived by the analysis of host-vector transmission dynamic models [33, 34]. These models assume the same SEI-SEIR epidemiological structure for both chikungunya and dengue: *Ae*. *albopictus* female adults may become infected after biting an infectious human host and start transmitting to other hosts after an incubation period and throughout the rest of their life (SEI). Human hosts may be infected by a bite of an infectious mosquito, develop symptoms after a latent period and may transmit to other mosquitoes for a given time, called infectious period (SEIR). The average number of mosquitoes infected by a single infectious human host in a population of fully susceptible mosquitoes and hosts is given by:

$$R_{HV}=k\;\beta_{V}\tau \frac{V}{H}\frac{\omega_{V}}{\omega_{V}+\mu_{V}}$$

where *k* is the biting rate of mosquitoes, β_V_ is the probability of transmission to mosquitoes per bite, τ is the infectious period of a human host, V is the density of female adult vectors, H is the density of human hosts, ω_V_ is the inverse of the mosquito incubation period and μ_V_ is the mosquito mortality rate. Similarly, the average number of hosts infected by a single infectious mosquito introduced in a population of fully susceptible mosquitoes and hosts is given by:

$$R_{VH}=k\;\frac{\beta_{H}}{\mu_{V}}$$

where β_H_ is the probability of transmission to humans per bite.

The basic reproduction number (i.e. the average number of secondary human infections caused by the introduction of a single infectious host in a population of fully susceptible mosquitoes and hosts) can be simply computed as the product of these two quantities:

$$R_{0}=R_{HV}R_{VH}$$

When a single infectious host is introduced in a population, the transmission process is stochastic and the host can heal before transmitting the infection even for values of R_0_ much above the epidemic threshold, purely because of chance. In this case, transmission dies out early without causing an epidemic outbreak. The probability that an outbreak will actually take place can thus be defined, and for a SEI-SEIR model is given by [35]:

$$p_{O}=1-\frac{R_{VH}+1}{R_{VH}(R_{HV}+1)}$$

We compute the reproduction numbers and probabilities of outbreak for both chikungunya and dengue in the ten study sites at different times of introduction of the index case. In particular, the values for the transmission probabilities, host infectious period and mosquito incubation period were taken from [28] for chikungunya, and from [36] for dengue. The mosquito incubation period and the transmission probabilities for dengue (but not for chikungunya) have been shown to depend on temperature [36], according to formulae initially estimated by Focks and colleagues on *Ae*. *aegypti* [37]. We used these formulae to compute those parameter values over time based on the daily temperature recorded at each trap locations. For both chikungunya and dengue, we assumed the same biting rate of 0.09 bites per mosquito per day estimated for the chikungunya outbreak occurred in 2007 in Italy [28]. The density of human hosts was estimated by dividing the census population of municipalities considered in the study by the corresponding urban surface. Finally, the density of female adult mosquitoes was obtained by dividing model estimates of the total population by the surface of the modeled capture area (about 56 ha, see S1 Text). Differences across sites in human density, local temperatures influencing epidemiological parameters, and the estimated vector abundance translate into a geographical heterogeneity in the risk of outbreaks.

## Results

### Model calibration

**Fig 2:**
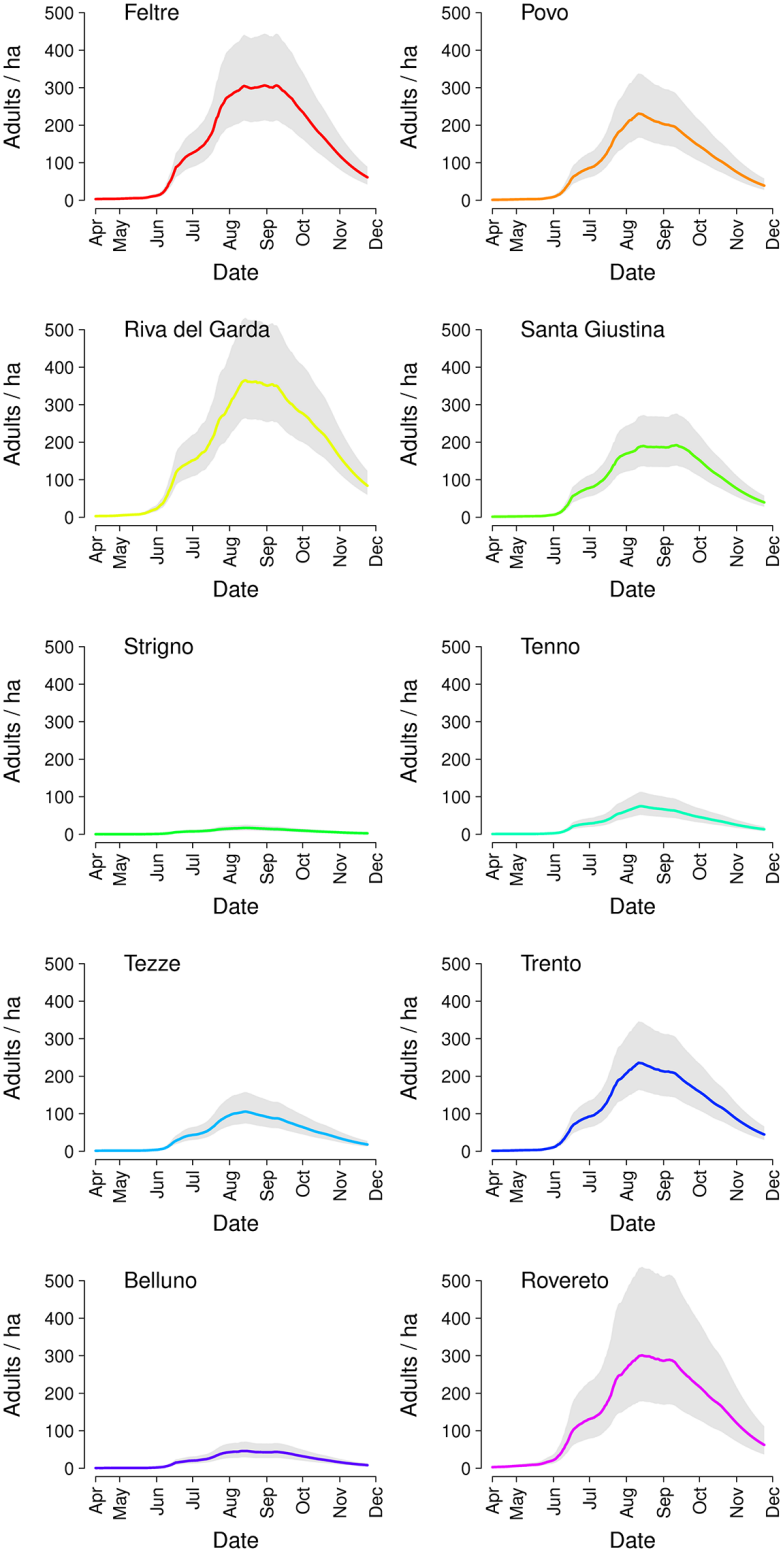
Model estimates for densities of adult females by study site; grey bars represent 95% CI calculated over 200 samples of the posterior distribution of parameters and 50 stochastic iterations.

In Fig 2, we show the density of female adults over time as estimated by the model for the different study sites. The curves are substantially synchronized across sites due to the relative homogeneity of temperature patterns over time, although quantitative differences in average temperatures (see S1 Text) contribute to the geographical heterogeneity in mosquito abundance. The average peak density ranges from 17.0 (95% CI: 10.3–25.5) adult female mosquitoes per hectare in Strigno to 365 (95% CI: 264–562) in Riva del Garda. These estimates were quite robust with respect to the model assumption on the initial number of eggs (see S1 Text).

**Fig 3:**
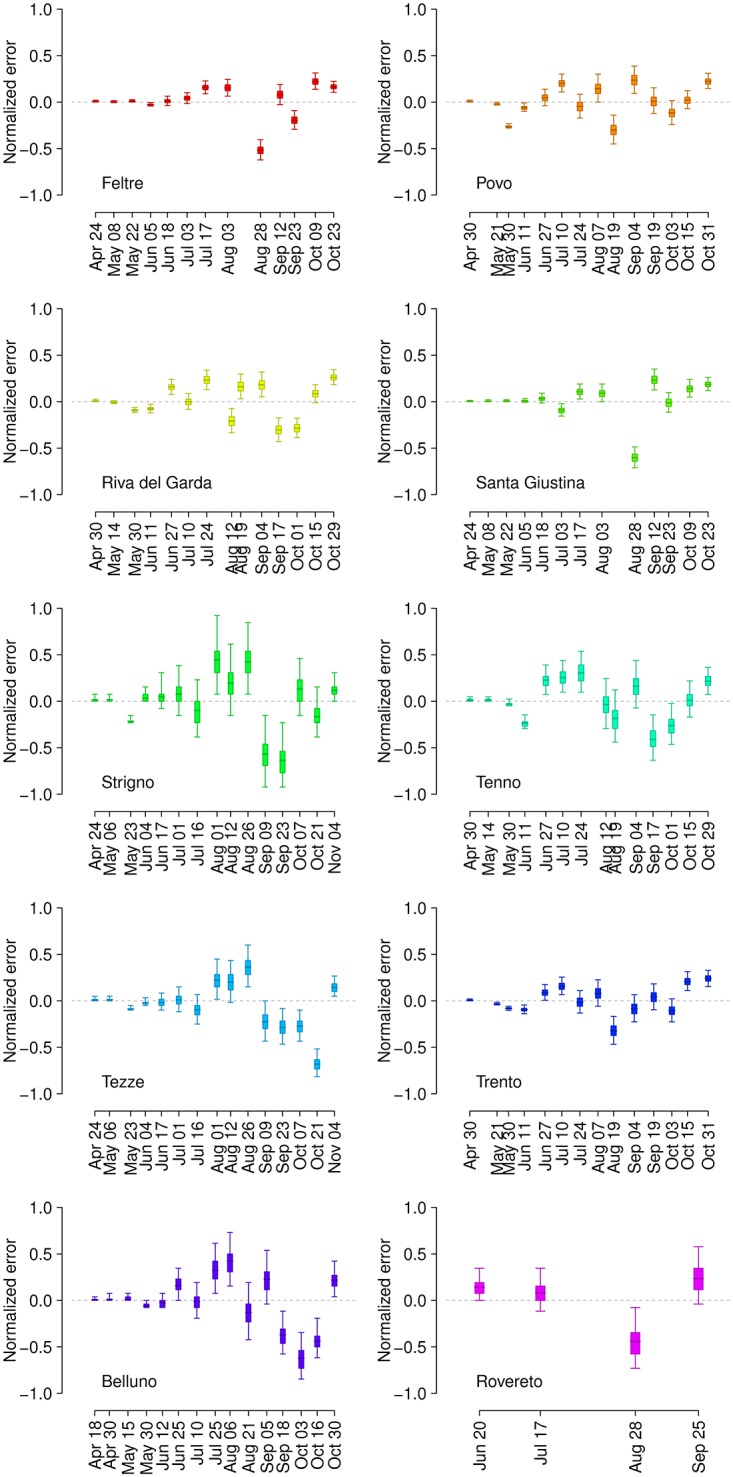
Boxplots of the errors between observed and simulated captures at each capture session in the ten study sites. The errors are normalized by the maximum number of captured females throughout the season.

Overall, the general seasonal pattern is well reproduced both qualitatively and quantitatively by the model (see S1 Text). Fig 3 shows boxplots of the distribution of errors between the observed number of captured females and corresponding model estimates (with 95% CI) for the ten study sites, normalized by the corresponding maximum number of captured females during the season to allow comparability across sites. In Table 1, we reported two metrics to assess the goodness of fit: the value of R^2^ between predicted and observed values and the root mean squared error, normalized by the maximum number of captured females (nRMSE). Both metrics are obtained by averaging over the 10,000 stochastic simulations. Two sites, Strigno and Belluno, are characterized by a lower R^2^ and higher nRMSE. The poorer fit at these two sites can be explained with the low estimated local mosquito abundance (see Fig 2), resulting in a stronger variability driven by stochastic effects in the capture process. Posterior means and 95% CI of estimates for the capture rate (α_0_) and the site-specific larval carrying capacities (a_s_), as estimated by the MCMC procedures, are reported in Table 2.

**Table 1:** Metrics for the goodness of fit between predicted and observed mosquito abundances.

Metrics	Feltre	Povo	Riva del Garda	Santa Giustina	Strigno	Tenno	Tezze	Trento	Belluno	Rovereto
**R**^**2**^	0.63	0.68	0.71	0.46	0.20	0.66	0.42	0.78	0.27	0.68
**nRMSE**	0.18	0.16	0.18	0.20	0.29	0.21	0.24	0.14	0.28	0.26

**Table 2:** Estimates of model parameters from the MCMC calibration procedure.

Parameter	α_0_	*a*_*s*_									
		Feltre	Povo	Riva del Garda	Santa Giustina	Strigno	Tenno	Tezze	Trento	Belluno	Rovereto
**Unit**	day^-1^	larval count									
**Mean**	7.7 10^−3^	95	66	73	77	7.6	36	63	55	20	52
**95% CI**	(4.9–10.4) 10^−3^	67–142	46–97	51–109	55–113	4.6–11.8	25–55	44–95	39–81	13–32	31–97

### Association between local factors, habitat suitability and transmissibility

**Fig 4:**
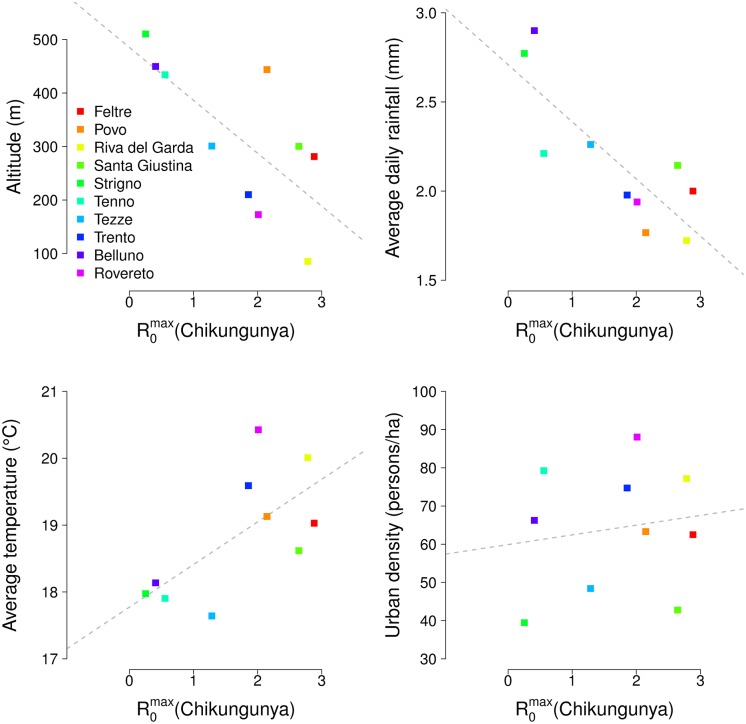
Scatterplots of the predicted peak of R_0_ for chikungunya at different sites and corresponding local variables; dashed lines represent regression lines.

**Fig 5:**
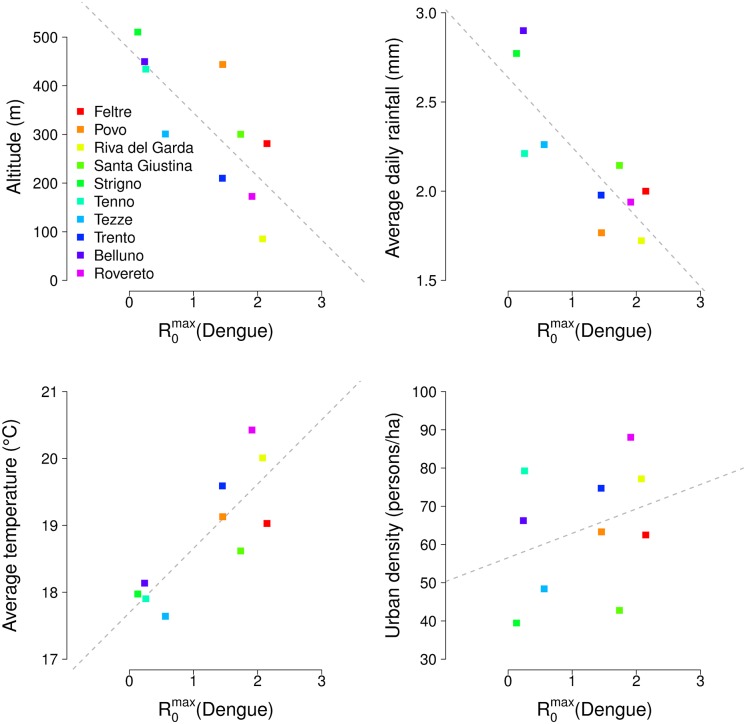
Scatterplots of the predicted peak of R_0_ for dengue at different sites and corresponding local variables; dashed lines represent regression lines.

A robust association between the average daily amount of precipitations and the estimated carrying capacities was found only in one time window, covering the period May 1^st^—June 5^th^, with a strong negative correlation (average Spearman’s ρ = -0.76; 95% CI: -0.69 –-0.82). When considering the number of rainy days, a negative correlation was also found for 17 time windows, covering periods between April 17^th^ and July 17^th^ (average ρ ranging between -0.70 and -0.84, depending on the time window, see S1 Text). Thus, late spring/early summer precipitations contribute negatively to local mosquito abundance and therefore to transmissibility of arboviruses. Figs 4 and 5 show the predicted peak value of R_0_ in the ten study sites for chikungunya and dengue respectively, against four site-specific variables: local altitude (computed as an average between the altitudes of active traps), average daily rainfall between May 1^st^ and June 5^th^, average temperature between April 1^st^ and October 31^st^, and urban density. These figures summarize the effects of different local environmental factors on the geographic heterogeneity in the transmissibility of arboviral diseases. A Spearman rank correlation test between the peak value of R_0_ for each disease and each variable was computed, and the results are summarized in Table 3. Lower altitudes and higher temperatures significantly increase R_0_ by creating favorable conditions for the vector population. Conversely, an excess of precipitations in late spring is associated with a reduction in the larval carrying capacity and, therefore, in the amount of mosquitoes available for transmission. Finally, urban population density does not seem to have a strong effect on the transmissibility of disease, despite the fact that this variable appears directly in the denominator of R_0_. This is explained by considering that, in a mountainous region such as the one under study, densely populated areas are concentrated at the bottom of the valleys, characterized by lower altitudes and warmer temperatures. Therefore, the negative effect of higher urban density on transmission is offset by the presence in the same sites of higher vector densities.

**Table 3:** Spearman’s correlation coefficients (ρ) between environmental variables and predicted peak of R_0_ for chikungunya and dengue.

Variables	Altitude		Average daily rainfall		Average temperature		Urban density	
	ρ	p-value	ρ	p-value	ρ	p-value	ρ	p-value
**R**_**0**_ **(chikungunya)**	-0.72	0.020	-0.82	0.004	0.67	0.034	0.16	0.667
**R**_**0**_ **(dengue)**	-0.77	0.009	-0.81	0.005	0.82	0.004	0.32	0.371

### Risk of arbovirus outbreaks

**Fig 6:**
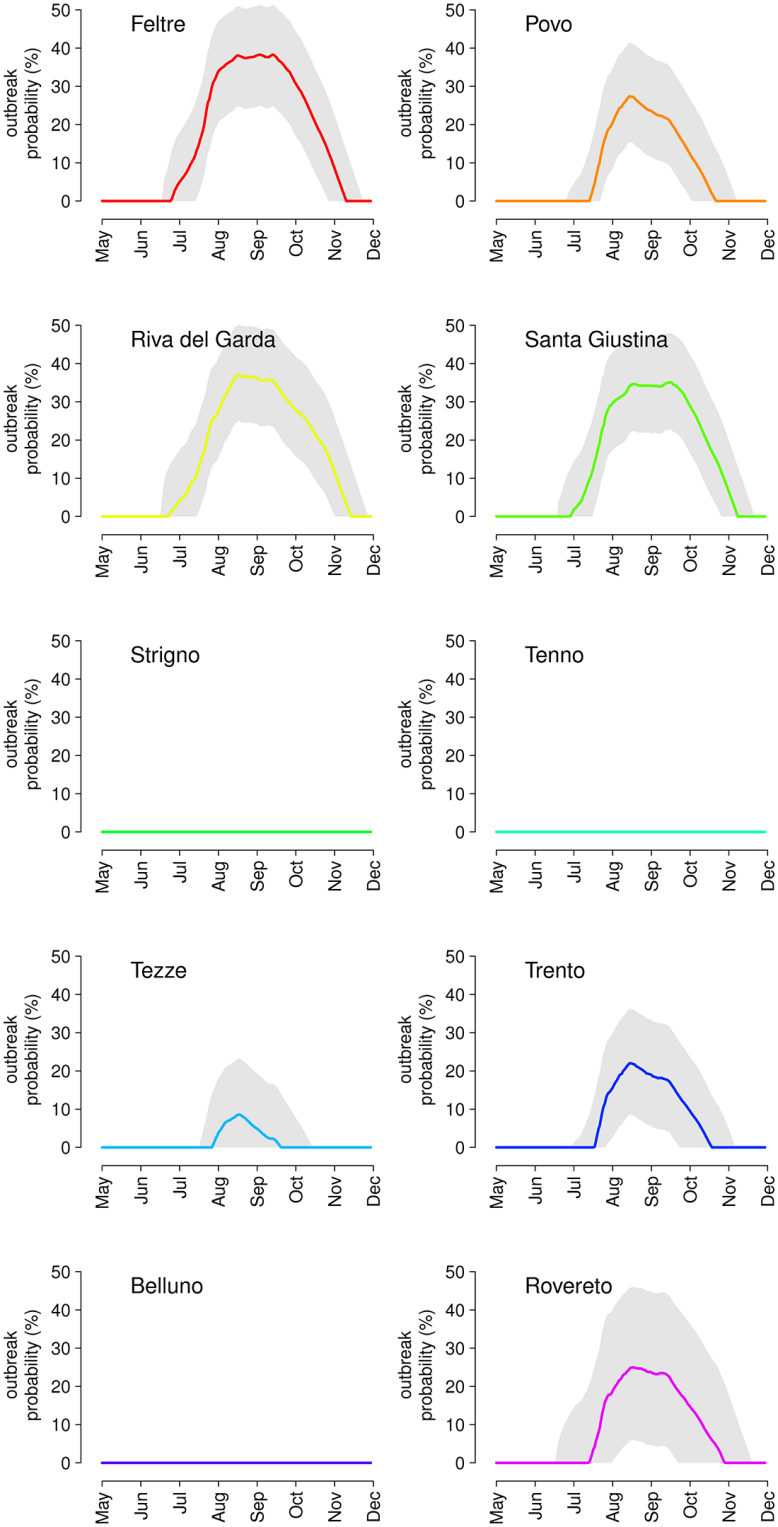
Model predictions for the probability of occurrence (average and 95% CI) of an outbreak of sustained chikungunya transmission caused by a single importation of an infected case occurred at different times of the year in the ten study sites.

We estimated the basic reproduction number of a potential chikungunya outbreak originated by a single imported case. The peak value of R_0_ is highly heterogeneous across sites, ranging from 0.25 (95%CI: 0.15–0.38) in Strigno to 2.9 (95% CI: 2.0–4.2) in Feltre and occurring, depending on the site, between mid-August and mid-September. The latter compares well with the R_0_ estimated for the chikungunya outbreak occurred in the Italian region of Emilia-Romagna in 2007 [28] and suggests the existence of further areas where similar outbreaks are likely to occur. Fig 6 reports the corresponding probability of observing an outbreak for different times of importation of the index case in the ten study sites. The three sites characterized by lowest estimated mosquito abundance (Strigno, Tenno and Belluno) are, according to the model, virtually safe from possible outbreaks. For all other sites, we can define a “chikungunya season” as the period over which the probability of observing an outbreak is higher than zero. The probability of an outbreak, averaged within each site’s chikungunya season, ranges from 4.9% in Tezze over a period of just below two months (end July to mid-September), to 25% in Feltre for a duration of 4.5 months covering the whole summer and up to mid-November. The peak probability within these seasons ranges from 8.6% (95% CI: 0.0–23.2%) in Tezze to 38.3% in Feltre (95% CI: 25.0–51.2%).

**Fig 7:**
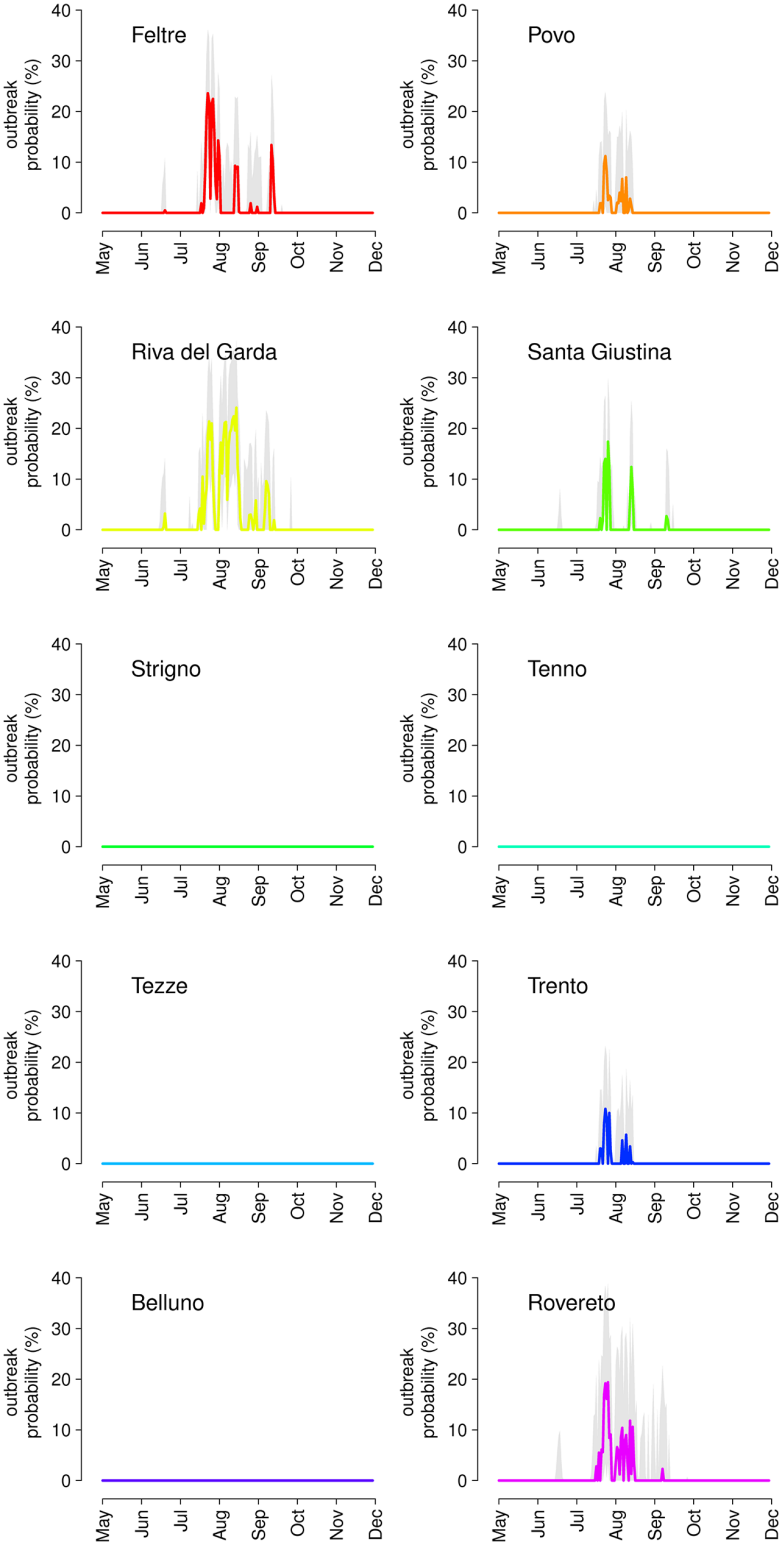
Model predictions for the probability of occurrence (average and 95% CI) of an outbreak of sustained dengue transmission caused by a single importation of an infected case occurred at different times of the year in the ten study sites.

Equivalent model predictions for dengue suggest generally lower values of R_0_ than for chikungunya. This is due to lower average transmissibility (confirmed experimentally in *Ae*. *albopictus* from northern Italy [38] and France [3]) and longer incubation periods [37, 38], resulting in longer generation times, so that a higher proportion of mosquitoes die while in the exposed (non-infectious) phase. Peak values of R_0_ range from 0.15 (95% CI: 0.09–0.22) in Strigno to 2.3 (95% CI: 1.7–3.3) in Riva del Garda and occur generally at the end of July, i.e. earlier than chikungunya. The anticipated peak of dengue transmissibility depends on the steady decrease of temperatures registered in 2014 after the end of July across all study sites (see S1 Text), which has reduced the estimated transmission probability and increased the incubation period [37], thereby offsetting the continued growth of vector abundance throughout August. Fig 7 shows the predicted probability of observing a dengue outbreak at different times of importation. Predictions are much more erratic over time than for chikungunya due to the strong temperature dependence assumed for dengue epidemiological parameters. Tezze adds to the list of virtually outbreak-free study sites, and the probability of outbreaks averaged over dengue seasons ranges from 4.2% in Trento, with a season length of one month across the end of July, to 10.8% in Riva del Garda over a two-month season that extends up to mid-September. The peak probability ranges from 12.1% (95% CI: 1.2–24.8%) in Trento to 27.8% (95% CI: 16.5–40.9%) in Riva del Garda.

### Spatializing the risk of outbreaks

**Fig 8:**
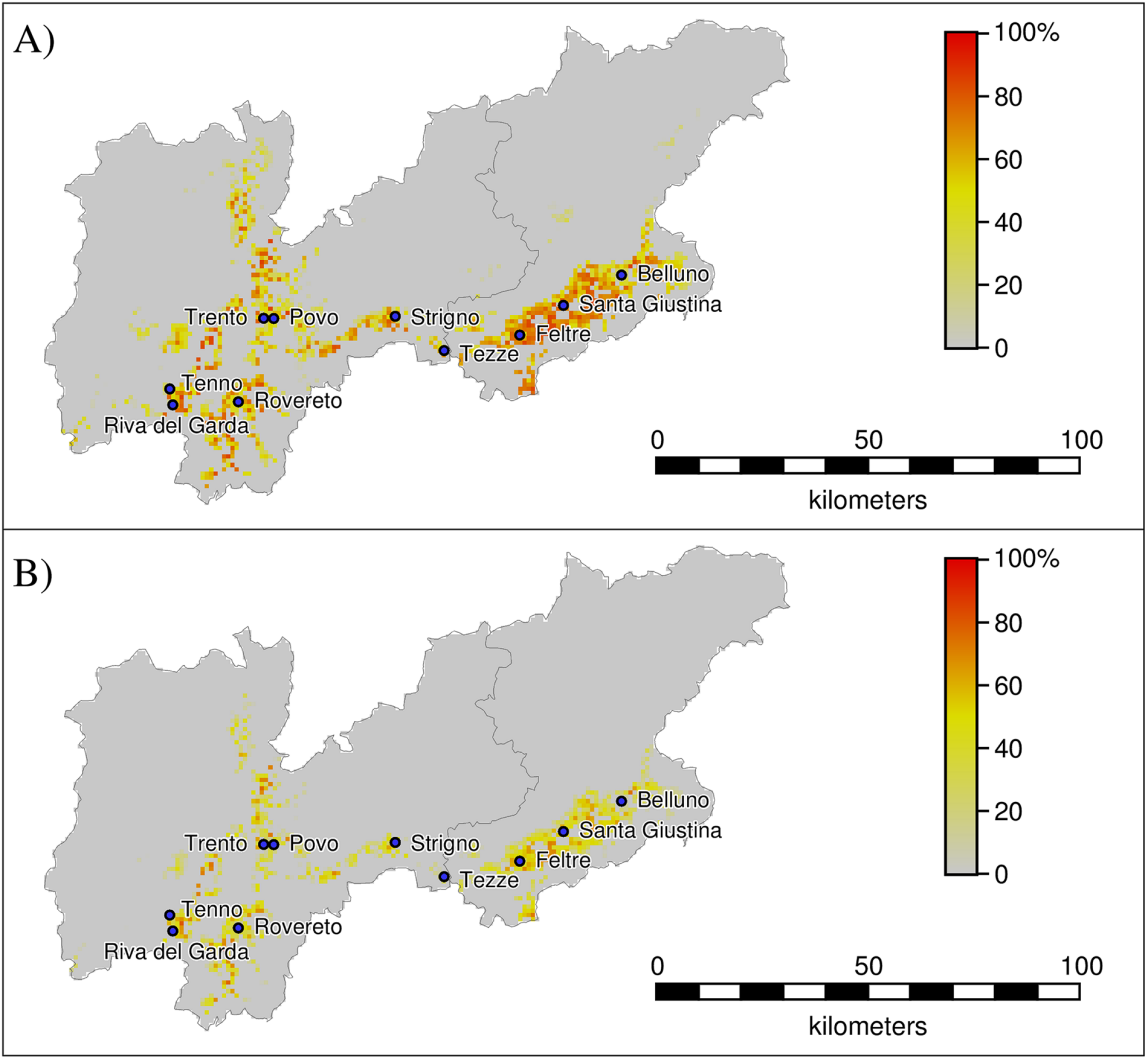
Peak values of the predicted outbreak risk over the study region under the maximum value of the larval carrying capacity estimated by our model in the 10 capture sites (a = 95). A) chikungunya; B) dengue.

A spatial interpolation of R_0_ is not possible, given the small number of data points (ten, i.e. one for each study site). For the same reason, a multivariate analysis to assess the contribution of environmental factors to local mosquito abundance or transmissibility would be statistically underpowered. Therefore, to provide spatial estimates of possible ranges of the outbreak risk, we applied our model to predict vector densities within the provinces of Trento and Belluno, based on local temperature records from satellite data and considering spatially constant values of the larval carrying capacity (see S1 Text). Afterwards, we computed the corresponding outbreak risks using population density maps derived from high-resolution census data. In Fig 8, we show the peak values of the outbreak risks for both infections in the worst-case scenario, i.e. using the maximum value of the larval carrying capacity estimated among our ten study sites. Fig 8 shows that areas with highest risk are rural villages placed at the bottom of the main valleys, where vector populations are predicted to be most abundant while human density is relatively lower. Minor valleys may be considered potentially free from the risk of outbreaks because of their lower mosquito abundance, which is in turn due to lower average temperatures. In the worst-case scenario, the fraction of the population living in areas with peak outbreak risk higher than zero is up to 74% of the total for chikungunya and 50% for dengue. However, the importation of a case in the study area at the exact time of the peak has a limited probability of generating an outbreak: on average 33% for chikungunya and 16% for dengue. The fraction of the population living in areas with a risk of outbreak higher than 50% after importation of a case at the time of highest risk is 25% for chikungunya and 6% for dengue. These results are in agreement with the specific findings obtained for the 10 study sites where entomological surveillance was carried out.

## Discussion

The diffusion of *Ae*. *albopictus* in temperate climates has created the conditions for local transmission of typically tropical pathogens, such as dengue and chikungunya viruses. These pathogens are continually imported into Europe through returning infected travelers from endemic areas, and may occasionally cause outbreaks of autochthonous transmission [7, 8, 10, 11]. In this work, we explore the probability of occurrence of dengue and chikungunya outbreaks in ten different sites from northern Italy, using a mosquito population model calibrated to capture data collected throughout 2014. The model was able to reproduce seasonal patterns in mosquito abundance, as well as variability across study sites. This geographic heterogeneity was represented in the model by local temperatures driving mosquito life cycles and by site-specific habitat suitability, summarized by a parameter encoding the larval carrying capacity. Increased habitat suitability was strongly associated with reduced precipitations in late spring and early summer. This negative correlation can be interpreted as a washout effect of rainfalls on breeding sites, previously observed in field experiments [39] and entomological observations [40], although other studies did not find any association between precipitations and *Ae*. *albopictus* abundance [41]. Given the strong inter-annual variability of precipitation patterns, our result will need to be validated by further studies.

Our analysis also provides estimates on the transmission potential and on the probability of outbreaks of chikungunya and dengue in the absence of control interventions, for different dates of importation of the index case. Based on model estimates of site-specific mosquito abundance and previous estimates for parameters driving host-vector transmission dynamics [28, 36], results suggest that six sites out of ten (Feltre, Povo, Riva del Garda, Santa Giustina, Trento and Rovereto) are affected by a high and sustained risk of chikungunya outbreak, as well as by a comparatively lower risk of dengue that extends over a shorter period. One study site (Tezze) had a negligible risk of a dengue outbreak but a significant risk of a chikungunya outbreak; the remaining three sites (Belluno, Strigno, and Tenno) were characterized by lowest observed abundances and had a negligible risk of outbreak for both diseases independently of the time of importation. Based on risk maps of potential outbreak in the study region, results suggest that the density of mosquitoes is expected to be higher in the main valleys. Within these areas, medium-sized towns (5,000–20,000 inhabitants) may be the local hotspots of an outbreak risk due to their lower urban densities compared to larger towns and cities. The limitation of these risk maps is the use of spatially-constant values for the parameter encoding the local availability and productivity of breeding sites. A punctual estimation of the actual outbreak risk in a given location would require additional entomological surveillance data similar to those collected from our 10 study sites. However, these maps are a useful tool for providing scenario predictions of the potential risk for Chikungunya and Dengue in our study region.

For what concerns the temporal variability of the risk of outbreak, it is interesting to compare our predictions with the observed distribution of the dates of importation in Italy in 2006–2014 ([42] and C. Rizzo, personal communication), which shows a clear seasonal pattern. For chikungunya, imported cases occur with equal probability throughout the year, except between June and September, which account for over 60% of imported cases overall. The peak of importations overlaps significantly with the peak of the outbreak risk for chikungunya (August and September, see Fig 6). Similarly, the rate of imported cases for dengue is about constant throughout the year, except for the months of August and September, representing alone about one sixth and one fourth of all imported cases respectively. In this case the peak of importations occurs slightly late with respect to the peak in outbreak risk (mid-July to mid-August, Fig 7), meaning that only a small fraction of imported cases occur when the risk of dengue is sufficiently high for an outbreak. This may explain why, despite the much higher number of imported cases of dengue (502 between 2006 and 2014) relatively to chikungunya (95 in the same period), Italy has already experienced a large chikungunya outbreak but only sporadic cases of autochthonous dengue transmission. However, these conclusions need to be taken with caution: the timing of importation of cases depends on international travels and on the timing of epidemics in countries from which the infection is imported. In addition, model predictions are strongly dependent on the recorded temperature patterns in 2014 in terms of the estimated mosquito abundance and, for dengue, also in terms of epidemiological parameters. Yearly variations in temperature records over time may provide different results for both the estimated risk and its timing. In the S1 Text, we propose a sensitivity analysis where a constant temperature variation between -2°C and +2°C is applied to the observed daily temperature. The analysis shows that model predictions are robust in terms of sites considered at risk of an outbreak, but the probability of outbreak and season length may vary considerably for sustained daily temperature variations higher than ±0.5°C.

Another key source of uncertainty for model estimates is the value of the biting rate. We assumed it to be equal to that estimated during the 2007 chikungunya outbreak [28] and performed a sensitivity analysis using boundaries of the estimated credible interval in the same study [28]. Resulting predictions range from complete absence of outbreak risk for both chikungunya and dengue in all sites, to a doubling of estimates of the peak probability of outbreak for both diseases with respect to the main analysis. However, even in the high biting rate scenario, sites with lowest abundances maintained a very low or negligible risk of chikungunya or dengue outbreaks. Full results of the sensitivity analysis are reported in the S1 Text.

In temperate climates, the population dynamics of *Ae*. *albopictus* during winters (overwintering) remains poorly understood [43]. Shortening photoperiods (duration of daylight) induce diapause in female adults, leading to generation of eggs that are particularly resistant to desiccation and cold [44]. It is thought that the hatching of diapausing eggs at low temperatures is slowed down, until warmer temperatures in spring allow survival of the larvae. However, other mechanisms have been shown to contribute to overwintering of *Ae*. *albopictus*, such as the availability of refuges with warmer microclimates for both adults and eggs [45], persisting oviposition at very low temperatures [43] and mosquito dispersal [46]. Lack of data on the abundance of vectors during winter in our study area prevents us from trying to reproduce the mechanism of persistence; therefore, the dynamics of mosquito abundance is simulated only within a single season. Although the population model for *Ae*. *albopictus* was able to reproduce well the mosquito capture data from the ten sites, further specifications on some of the biological modifications of the mosquito life cycle at low temperatures and short photoperiods may improve model accuracy. For example, the model has a general tendency to underestimate the sharp drop in the observed number of captured females between the end of October and the start of November. One reason for this may be that the model neglects the reduced developmental rates [47] and increased durations of gonotrophic cycles [48] caused by diapause. In addition, we may be underestimating adult mortality rates at temperatures below 10°C [49], since our mortality curves are based on data collected at a minimum temperature of 15°C [15].

Finally, a general limitation of population dynamics models applied to arbovirus vectors is the lack of biological data for the endemic mosquito strain under local, possibly natural, environmental conditions [50]. This limitation applies also to this work, where parameters for the mosquito life cycle were estimated from laboratory experiments on *Ae*. *albopictus* from Reunion [15]. Collection of analogous data from strains adapted to temperate areas is therefore needed to improve the accuracy of model estimates on vector abundance. As for epidemiological parameters, we used estimates from the 2007 chikungunya outbreak in Italy [28] and from the 2014 dengue outbreak in Madeira [36]. Although the latter epidemics was spread by *Ae*. *aegypti*, there are no available estimates for actual outbreaks caused by *Ae*. *albopictus*. We preferred to avoid using data from laboratory experiments on *Ae*. *albopictus* [3], given the uncertainties in extending these findings to natural conditions. The competence of *Ae*. *albopictus* for transmitting dengue is generally considered lower than that of *Ae*. *aegypti*, the dominant vector of these diseases in endemic areas [51]. However, recent experimental results on mosquitoes from southern France [3] found a transmission efficiency for dengue of up to 16–28% in *Ae*. *albopictus* and up to 15–18% in *Ae*. *aegypti*; however the shorter extrinsic incubation period in the latter species may compensate the lower transmission efficiency in terms of epidemic potential [3]. The high vectorial competence of *Ae*. *albopictus* from temperate climates suggests the possibility that a rapid evolutionary adaptation towards a higher transmissibility is ongoing [3,4]; in this case the risk of arboviral diseases would be higher than estimated. Furthermore, the Madeira outbreak demonstrated that *Ae*. *aegypti* poses an additional serious threat to public health [36] in Europe. *Aedes aegypti* has recently re-established also on the European mainland (namely in territories surrounding the Black Sea) after an absence of several decades [52, 53]. International travel of persons and goods increases episodes of introduction of *Ae*. *aegypti* in other areas, as documented in the Netherlands [52]. Entomological surveillance and mosquito control are therefore critical to prevent its re-establishment, and the ensuing costs to health systems [44], in other European countries with a suitable climate [54].

Medical surveillance against arboviral diseases has been introduced in Italy since the chikungunya outbreak in the Emilia-Romagna region in 2007 [7]. When a case is diagnosed during the mosquito season, a number of preventive measures are applied, including quarantine of the infected individuals and vector control interventions (e.g. adulticide spraying in combination with larvicide treatment of public catch basins) in the neighborhood of the imported case’s residence. Furthermore, many local authorities in areas heavily infested by *Ae*. *albopictus* have introduced routine vector control to reduce the transmission risk even in the absence of imported cases. Thanks to preventive and reactive interventions, autochthonous transmission of arboviral diseases has not been observed in Italy since the 2007 outbreak, despite the high risk estimated by our model and the high importation rates (48 chikungunya and 78 dengue cases between May and October 2014 alone). This public health success highlights once again the importance of surveillance and preventive actions against arboviral diseases in temperate climates.

There are other arbovirus infections for which *Ae*. *albopictus* is competent and which may be of great public health interest. *Aedes albopictus* can enhance the spread of West Nile Virus [55], which is mainly transmitted by endemic mosquito species such as *Culex pipiens* L. [56]. Importation of Zika virus infection [57] raises concerns as the pathogen, which generally causes mild symptoms, is suspected of being associated with Guillain-Barre’ syndrome, a severe autoimmune neurologic disease [58], and with congenital microcephaly, a disabling and potentially lethal condition in newborns [59]. We did not include in our analysis the risk of outbreak for West Nile Virus because of the significant complications in the transmission dynamics [55] due to the presence of other animal hosts such as avian species and of other vector species such as *Cx*. *pipiens*. Furthermore, we did not consider the potential risk of Zika virus outbreaks, given that estimates of epidemiological parameters from actual outbreaks [59–61] are not yet available for this infection.

The continued expansion and environmental adaptation of *Ae*. *albopictus* is making chikungunya, dengue and other tropical diseases a growing threat for public health authorities in temperate climates. Expected trends in climate and land use and more frequent pathogen introductions due to worldwide intensification of travels will exacerbate this problem in the years to come. For example, the “explosive epidemic” of Zika virus ongoing in Brazil [59] poses specific concerns related to the massive inflow of international tourists expected during the upcoming Olympics in Rio de Janeiro in the second half of August. The return of infected travelers to countries in the northern hemisphere, where the mosquito season will be at its peak, may facilitate the geographical spread of Zika virus. Enhanced entomological and medical surveillance will be especially important to prevent such a scenario; population and transmission dynamic models can assist surveillance policies by quantifying the risks of arboviral infections, designing effective intervention strategies and optimizing resource allocation for the control of *Ae*. *albopictus* populations.

## Supporting Information

S1 Dataset Capture data from the ten municipalities. For each date and municipality, the number of active traps and the total number of female *Ae*. *albopictus* captured is reported.
(CSV)

S1 Text Supplementary Material. Further details on model implementation, validation and sensitivity analyses.
(PDF)
