# Supplementary material for: Potential Risk of Dengue and Chikungunya Outbreaks in Northern Italy Based on a Population Model of Aedes albopictus (Diptera: Culicidae)
Source: PLoS Negl Trop Dis. 2016 Jun 15;10(6):e0004762. doi: 10.1371/journal.pntd.0004762 (PMC4909274; doi:10.1371/journal.pntd.0004762)
Supplement: S1 Text — Further details on model implementation, validation and sensitivity analyses. (PDF) [file pntd.0004762.s002.pdf]

SUPPLEMENTARY MATERIALS

Potential risk of dengue and chikungunya outbreaks in northern Italy based on a population model of Aedes albopictus (Diptera: Culicidae)

G. Guzzetta, F. Montarsi, F. Baldacchino, M. Metz, G. Capelli, A. Rizzoli, A. Pugliese, R. Rosà, P. Poletti, S. Merler

Population model

We developed an Ordinary Differential Equations (ODE) model representing vital dynamics for a population of *Aedes albopictus* and fitted it to capture data from 10 study sites. State variables in the model represent the number of individuals in each developmental stage of the mosquito (egg, larva, pupa, female adult). Each stage is characterized by two temperature-dependent rates: mortality and progression to the next developmental stage for eggs, larvae and pupae; and mortality and egg deposition for female adults. The rate of egg deposition reproduces the gonotrophic cycle, and each female adult is assumed to depose a fixed average number of eggs at each cycle. In addition, female adults can be captured at a given rate, being thereby removed from the population. A site-specific carrying capacity is assumed for the mortality rate at the larval stage, representing the site-specific habitat suitability. Equations for the model are reported hereafter:

$$\begin{cases} \dot{E} = n_E d_V V - (m_E + d_E)\, E \\ \dot{L} = d_E E - \left(m_L \left(1 + \dfrac{L}{a_s}\right) + d_L\right) L \\ \dot{P} = d_L L - (m_P + d_P) P \\ \dot{V} = \dfrac{1}{2} d_P P - (m_V + \alpha_s(t))\, V \end{cases}$$

where:

- E, L, P and V represent the number of eggs, larvae, pupae and female adults respectively;
- n_E is the average number of deposed eggs per female adult per oviposition;
- d_V is the temperature-dependent rate of egg deposition for female adults;
- d_E, d_L and d_P are the temperature-dependent rates of progression to the following developmental stage; the coefficient ½ in the equation of female adults accounts for the sex ratio in the development of pupae into adults.
- m_E, m_L, m_P and m_V are temperature-dependent mortality rates for each stage;
- a_s is the site-specific larval carrying capacity;
- $\alpha_s(t)$ is the capture rate for female adults and models the capture process. In the capture experiment, traps are displaced at much larger distances than the mosquito flight range [1] so that each trap targets a mosquito population that is disjoint from all others. A different number of traps was deployed in different sites, and not all traps were active at all times. Therefore, the capture experiment targets a mosquito population covering an area that changes by site and over time. To overcome this complication, we model a mosquito population living over a total area covered by 8 traps, which is the maximum number of traps deployed in the same site within the experiment. Assuming a flight range of r=150m (in line with literature estimates [1]),

the total area covered by the model population is $A = 8\pi r^2 = 56.55$ ha. We define a maximal capture rate α_0 as the fraction of the total modeled population that is captured per day when all 8 traps are active. α_0 was a free model parameter, calibrated against data. The actual capture rate α_s(t) at each site and session is obtained by scaling α_0 by the fraction of the modeled area covered by active traps, which is given by the number of active traps at each session and site divided by 8.

Calibration procedure

The model population is initialized at April 1st with all state variables set to zero except for a fixed, large number of initial eggs (N_E = 10,000), subject to sensitivity analysis (see below); each day, the value of temperature-dependent rates are recomputed on the basis of the average daily temperature registered by traps at each study site. The functional forms expressing the temperature dependence of the 8 rates, estimated in a previously published study [2] using experimental data [3], are reported below with corresponding parameter values.

$$d_E(T) = \frac{1}{p_{E1} - p_{E2} e^{-\left(\frac{T-20}{p_{E3}}\right)^2}}$$

$$d_L(T) = \frac{1}{p_{L1}T^2 + p_{L2}T + p_{L3}}$$

$$d_P(T) = \frac{1}{p_{P1}T^2 + p_{P2}T + p_{P3}}$$

$$d_V(T) = \frac{1}{p_{V1}T^2 + p_{V2}T + p_{V3}}$$

$$\mu_E(T) = q_{E1} - q_{E2} e^{-\left(\frac{T-25}{q_{E3}}\right)^6}$$

$$\mu_L(T) = q_{L1} + q_{L2} e^{(T-q_{L3})}$$

$$\mu_P(T) = q_{P1} + q_{P2} e^{(T-q_{P3})}$$

$$\mu_V(T) = q_{V1} + q_{V2} e^{(T-q_{V3})}$$

p_{E1}	p_{E2}	p_{E3}	q_{E1}	q_{E2}	q_{E3}
6.9	4.0	4.1	506	506	27.3
p_{L1}	p_{L2}	p_{L3}	q_{L1}	q_{L2}	q_{L3}
0.12	-6.6	98	0.029	858	43.4
p_{P1}	p_{P2}	p_{P3}	q_{P1}	q_{P2}	q_{P3}
0.027	-1.7	27.7	0.021	37	36.8
p_{V1}	p_{V2}	p_{V3}	q_{V1}	q_{V2}	q_{V3}
0.046	-2.77	45.3	0.031	95820	50.4

A graphical representation of the development and mortality rates for different values of T is reported in [2], Figures 2b and 2d.

The maximal capture rate α_0 and the 10 site-specific larval carrying capacities, a_s, were free model parameters. The predicted cumulative number of captured females over each capture session was fitted to the observed one by using the Poisson likelihood, defined as:

$$L(\boldsymbol{O}, \boldsymbol{p} | \alpha_0, \mathbf{a}) = \prod_s^S \prod_j^{N_s} \frac{e^{-p_{sj}(\alpha_0, a_s)} p_{sj}(\alpha_0, a_s)^{O_{sj}}}{O_{sj}!}$$

where:

- O_{sj} is the observed number of captured females during session *s* at study site *j*;
- p_{sj} is the model-predicted number of captured females by session and study site, dependend on free model parameters;
- α_0 is the maximal capture rate (assumed equal for all sites);
- a_s is the site-specific larval carrying capacity;
- S is the number of study sites ($S = 10$)
- N_s is the number of capture sessions for site *s*.

We used uniform priors on parameters (constrained to positive values) and computed their posterior distributions by means of a Monte Carlo Markov Chain (MCMC) approach, using random-walk Metropolis-Hastings sampling with normal jump distributions. Convergence of MCMC (1 million iterations) was assessed by considering several different starting points and by visual inspection, after a burn-in period of 10000 iterations. After calibration, results were computed by running a stochastic implementation of the same model, using $M = 200$ parameter values sampled from the posterior distributions estimated by the MCMC procedure and repeating 50 iterations per parameter set.

Comparison of observed and modelled captures

Figure S1 compares the observed (black squares) and modelled (in red; average and 95%CI) numbers of adult females captured at each session in the 10 study sites. The numbers of captured females reported depend on the number of active traps at a given site and capture session (see Supplementary Dataset); therefore, they do not directly reflect the actual abundance of female mosquitoes in the site. Consequently, the ranking of sites with respect to density (shown in Figure 2 in the main text) does not necessarily reflect the ranking with respect to the number of captures. The figure shows that the seasonal pattern is correctly reproduced by the model. Sites with a low R^2 between average model predictions and data (Strigno and Belluno, see Table 1 in the main text) are those with the largest model-predicted variability, due to the higher impact of stochastic effects in the capture process when mosquito abundance is low.

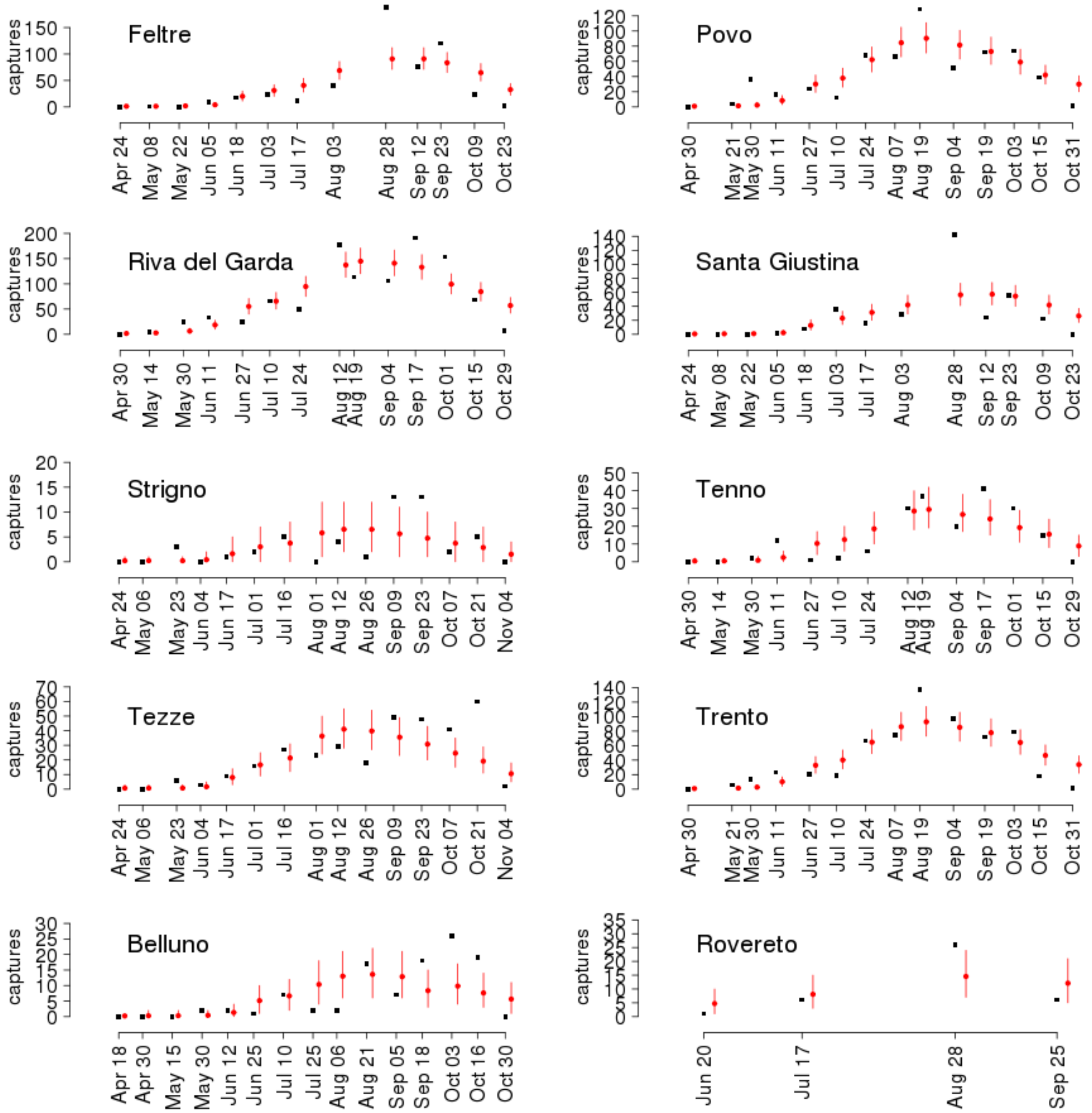

Figure S1: comparison between observed and modeled captures over time for the ten study sites

Temperature over time

Figure S2 reports the temperatures for each study site, calculated as the average across temperatures measured at the trap locations. There is a considerable homogeneity in temperature patterns, attributable to the limited geographic area covered by the study, which results in similar temporal patterns for the mosquito abundance across sites. However, quantitative temperature differences exist, with a daily temperature variation across sites ranging from 1.5 to 6.7°C, and a mean yearly temperature ranging from 13.2 in Strigno and Tezze to 15.4 in Rovereto. These variations, together with site-specific

larval carrying capacity values, explain the geographic variability found in the observed vector abundance and reproduced by the model.

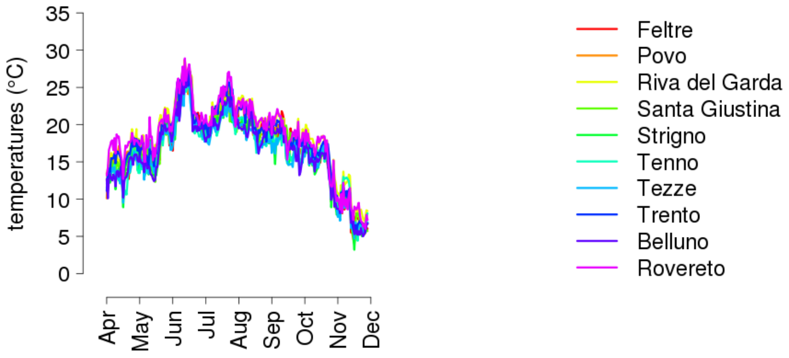

Figure S2: observed temperatures over time for the ten study sites

Sensitivity of model with respect to the initial number of eggs, N_E

The number of eggs at the beginning of the mosquito season is not known, therefore it is important to verify that the model predictions are robust with respect to the corresponding assumed value, $N_{E,}$ set to 10,000 in the main analysis. To this aim, we run the model with values of N_E going from 100 to 100,000,000. Using a value of N_E = 100 resulted in the systematic extinction of the mosquito population in some sites; therefore this value was discarded as it was not compatible with the observed presence of mosquitoes at all sites during the considered season. For N_E = 1,000, stochastic extinctions occurred in up to 17% of simulations, depending on the site; for $N_E \geq$ 10,000 stochastic extinctions did not occur. The model-predicted number of eggs at all sites and for all values of N_E never exceeded 700,000 even when using N_E = 100,000,000; therefore, we restricted our sensitivity analysis to values of N_E between 1,000 and 1,000,000 eggs.

When using N_E = 1,000, a slightly lower peak mosquito abundance was found with respect to the baseline (N_E =10,000), with a site-dependent relative difference between 7% and 19%. However, it is to be noted that stochastic effects (signaled by the extinction of the population in some simulations for N_E = 1,000) may have inflated differences in predicted values in some sites. For $N_E \geq$ 10,000, maximum differences in the estimated mosquito density with respect to the baseline did not exceed 15% at any study site (see Figure S3). Corresponding variations from the baseline of the predicted risk were also limited, with average values across sites ranging from 4.5% with N_E = 1,000 to 2.5% with N_E = 1,000,000.

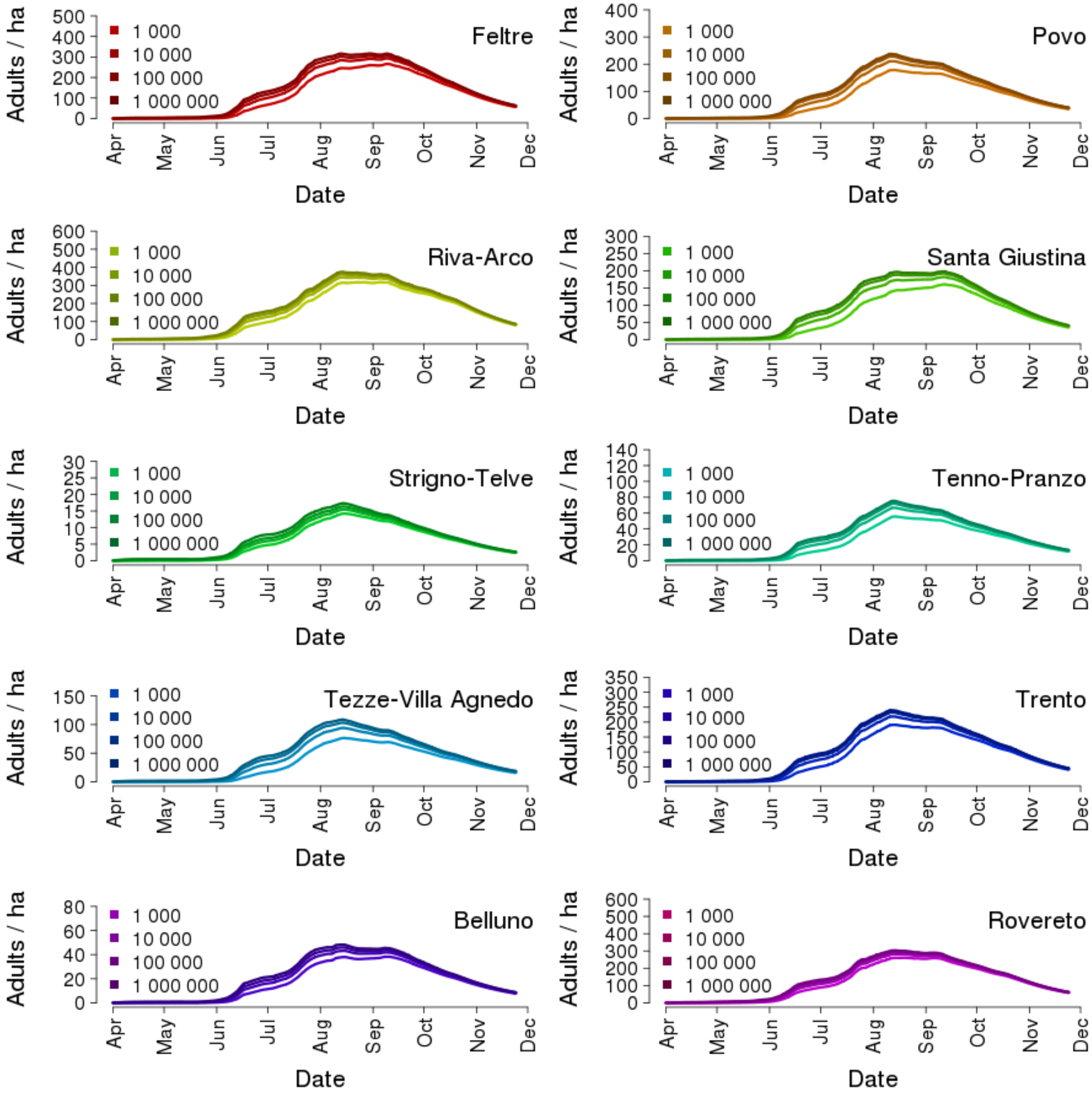

Figure S3. Predictions of mosquito density by different values of the initial number of eggs.

Association between local factors and habitat suitability

Table S1 reports the time windows for which a significant correlation (Spearman) was found between the estimated value of carrying capacity and local precipitations.

Precipitation variable	*Time window*	*Duration (weeks)*	*Spearman's ρ (95% CI)*
total precipitations	May 1st - June 5th	5	-0.76 (-0.69 – -0.84)
number of rainy days	April 17th – July 3rd	9	-0.70 (-0.64 – -0.79)

May 8th – July 3rd	8	-0.73 (-0.66 – -0.81)
May 15th – June 26th	6	-0.73 (-0.66 – -0.80)
May 15th – July 3rd	7	-0.81 (-0.75 – -0.87)
May 15th – July 10th	8	-0.78 (-0.72 – -0.84)
May 22nd – June 19th	4	-0.72 (-0.65 – -0.80)
May 22nd – June 26th	5	-0.74 (-0.68 – -0.81)
May 22nd – July 3rd	6	-0.81 (-0.75 – -0.87)
May 22nd – July 10th	7	-0.79 (-0.73 – -0.84)
May 22nd – July 17th	8	-0.71 (-0.65 – -0.79)
May 29th – June 26th	4	-0.73 (-0.66 – -0.79)
May 29th – July 3rd	5	-0.80 (-0.74 – -0.86)
May 29th – July 10th	6	-0.78 (-0.72 – -0.83)
May 29th – July 17th	7	-0.70 (-0.64 – -0.78)
June 5th – July 3rd	4	-0.84 (-0.78 – -0.88)
June 12th – July 10th	4	-0.75 (-0.69 – -0.80)

Table S1: correlation of precipitations with carrying capacities in selected time windows

Model estimates for R_0 by date of case importation

Figure S4 and S5 report estimates of R_0 by date of importation of the initial case for Chikungunya and dengue. Summary information on peak and average values are reported in the main text.

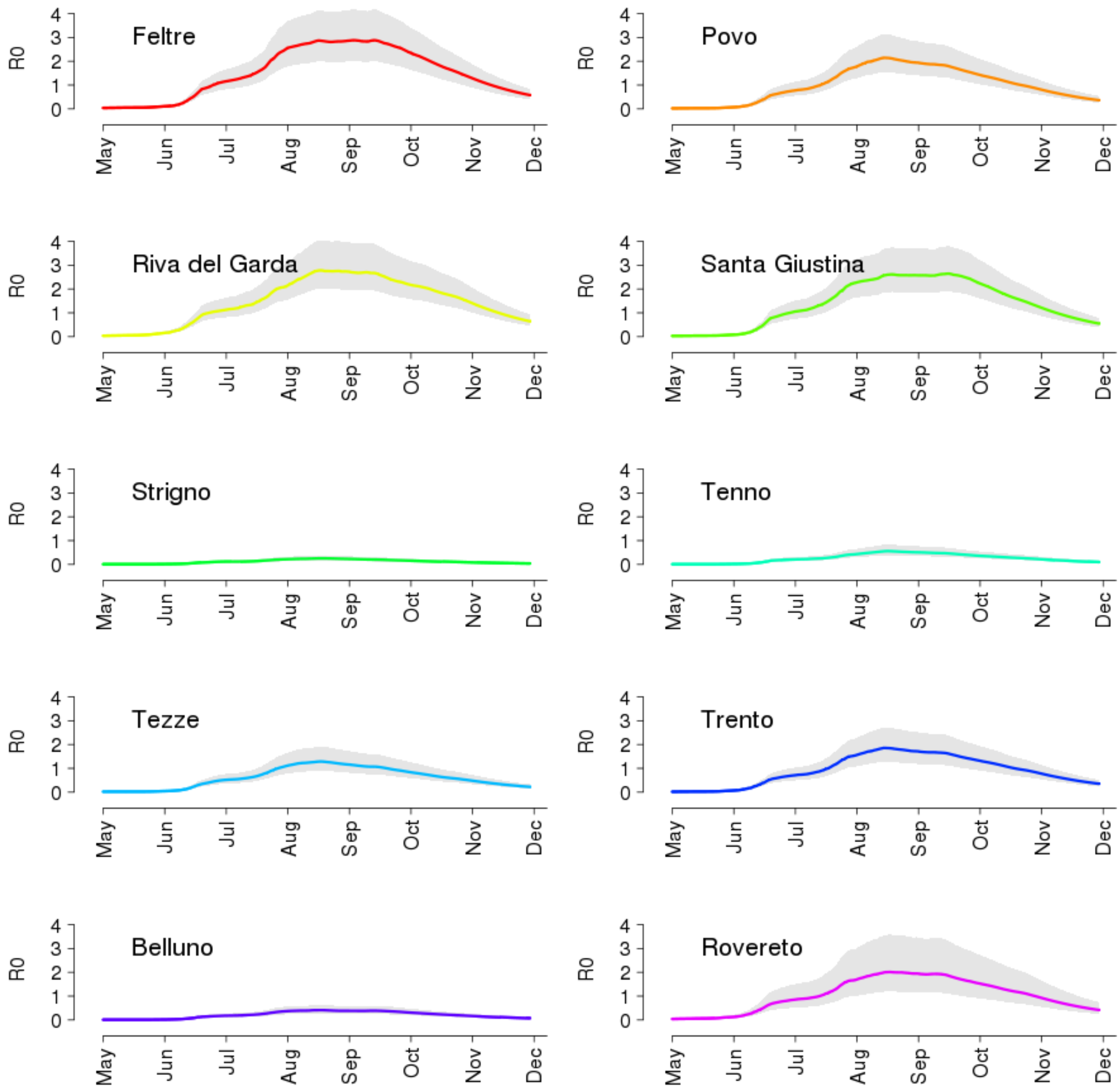

Figure S4. Model predictions for the basic reproduction number (average and 95% CI) of a Chikungunya outbreak caused by a single importation of an infected case occurred at different times of the year in the ten study sites.

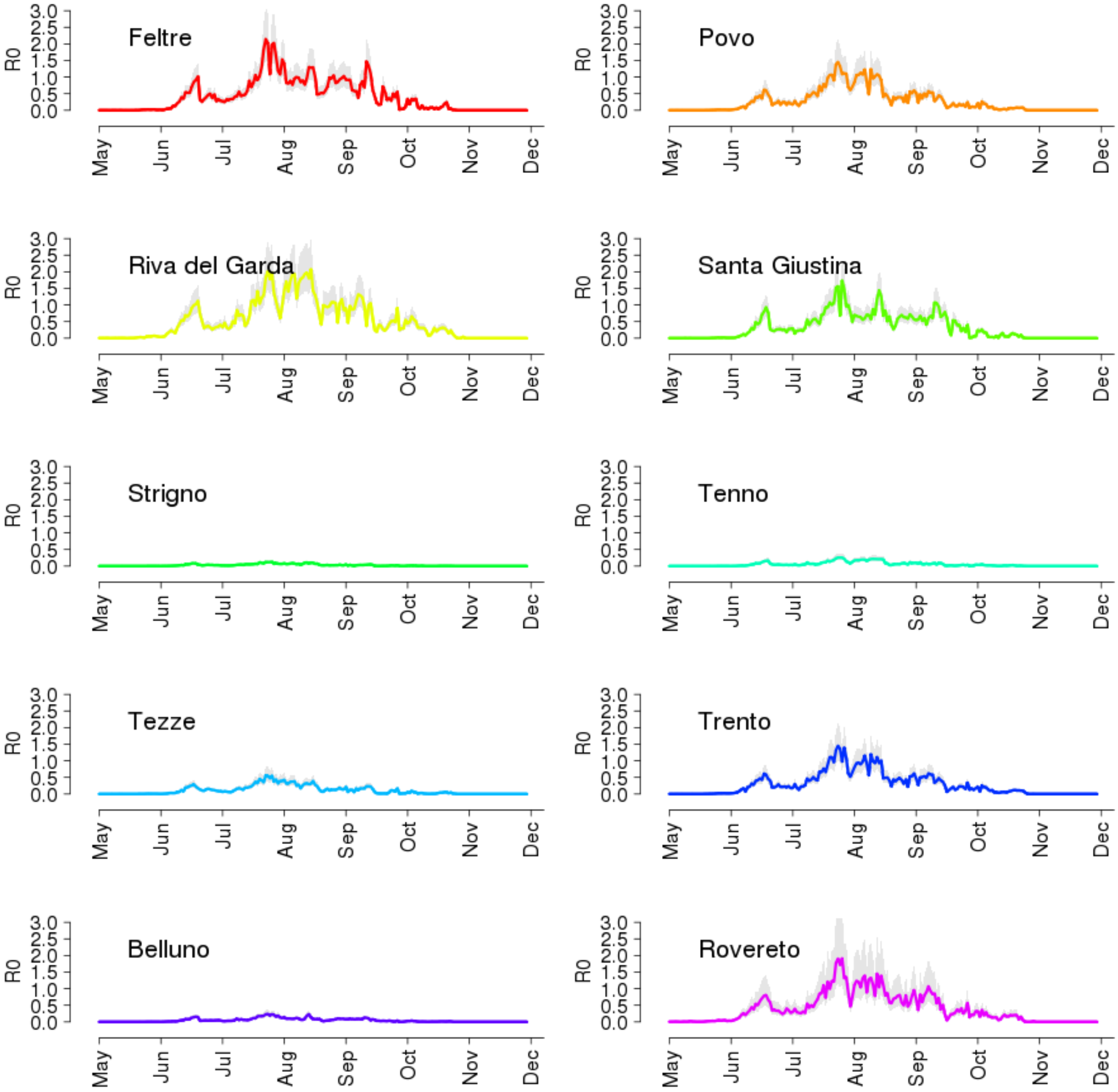

Figure S5. Model predictions for the basic reproduction number (average and 95% CI) of a dengue outbreak caused by a single importation of an infected case occurred at different times of the year in the ten study sites.

Sensitivity analysis with respect to temperature

In this section, we evaluate the sensitivity of model predictions with respect to the observed temperature. In particular, we re-compute the site-specific vector abundance using daily temperature values increased or decreased by a constant variation (chosen between -2°C and +2°C in different scenarios); we then use new estimates of vector abundance to determine the probability of outbreak. In Figures S6 and S7 we show the variation with temperature of the length of the Chikungunya season

(defined, as in the main text, as the period of the year with a higher than zero probability of outbreak) and of the mean outbreak probability computed over the season. A temperature drop by 2 degrees would make the risk of Chikungunya negligible everywhere except in Feltre and Riva del Garda (where it would however be very low and for a very short season). On the other hand, an equivalent temperature increase would include in the list of sites at risk only one additional site (Tenno), with a low probability; however, under this hotter temperature scenario the mean risk of outbreak would about double in almost all cases, with a moderate increase in season length as well.

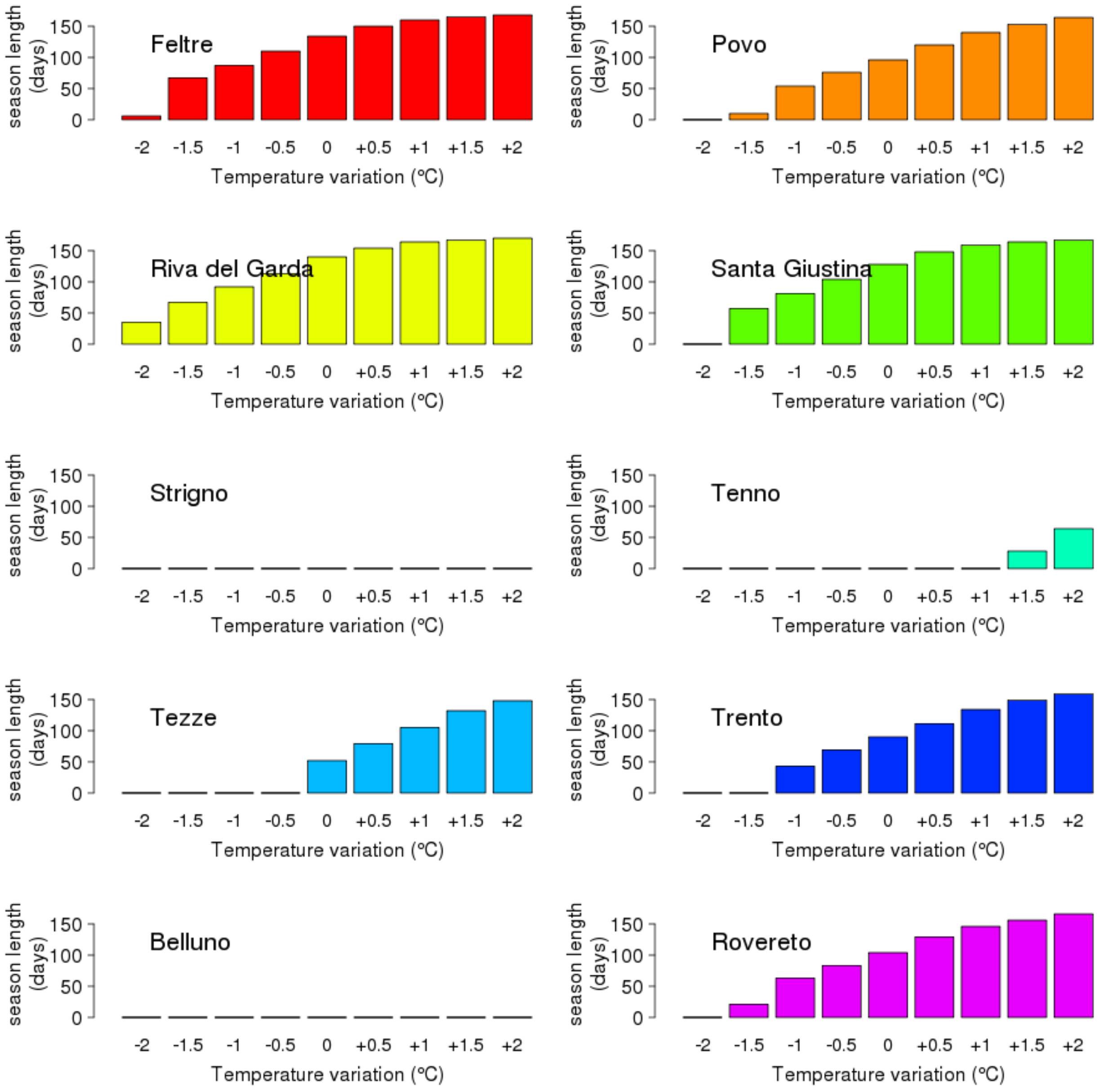

Figure S6: predicted season length for Chikungunya with respect to changes in daily temperature

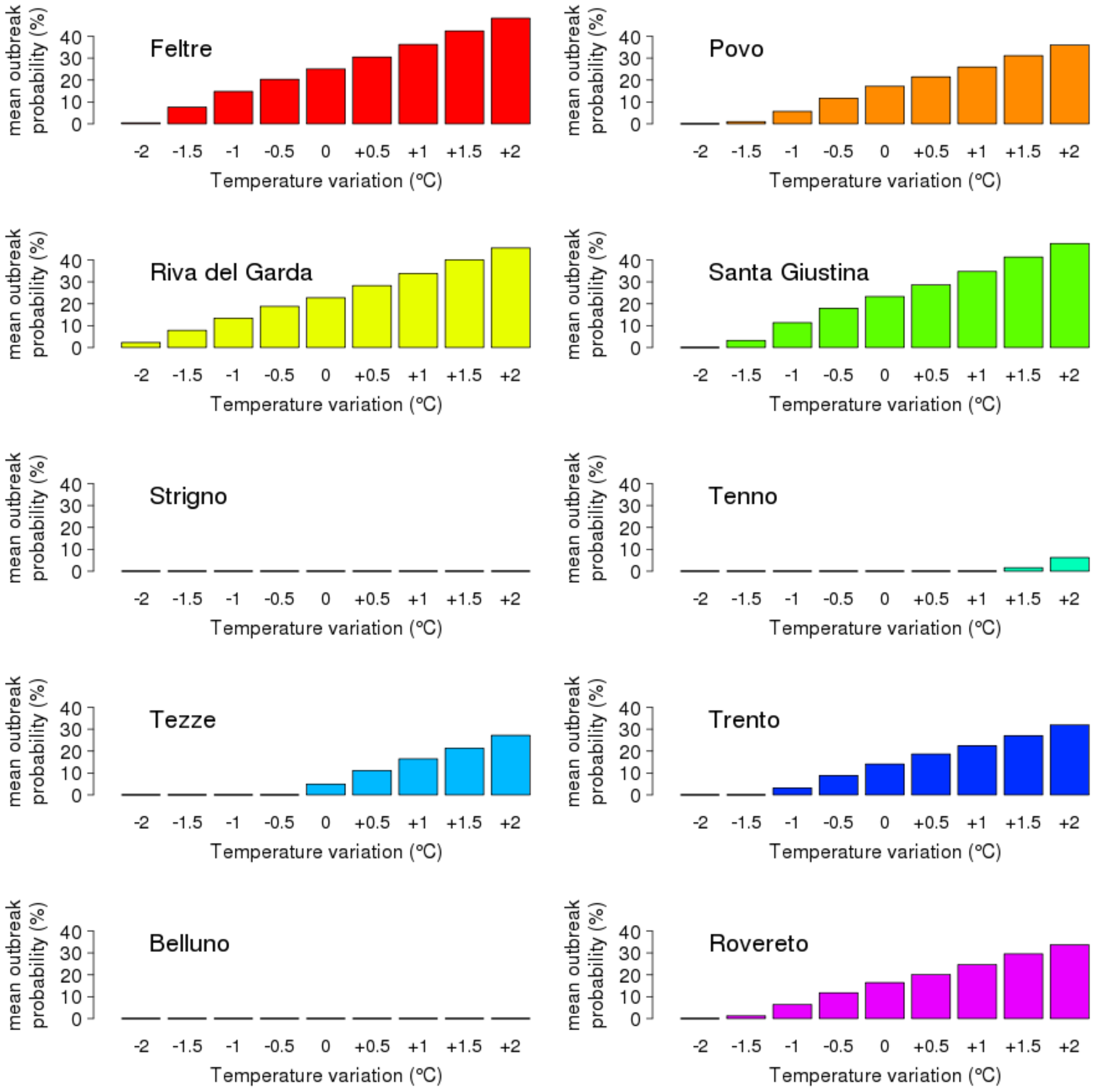

Figure S7: predicted mean probability of a Chikungunya outbreak with respect to changes in daily temperature

Figures S8 and S9 show analogous results for dengue. In this case, a daily temperature drop of 1.5 degree is sufficient to eliminate the risk of outbreak in all sites, while an increase by 2 degrees would add only Tezze to the list of at-risk sites. However, in this warmer scenario, the average probability would double in all sites at risk and the season length would be extended by 2 to 4 times.

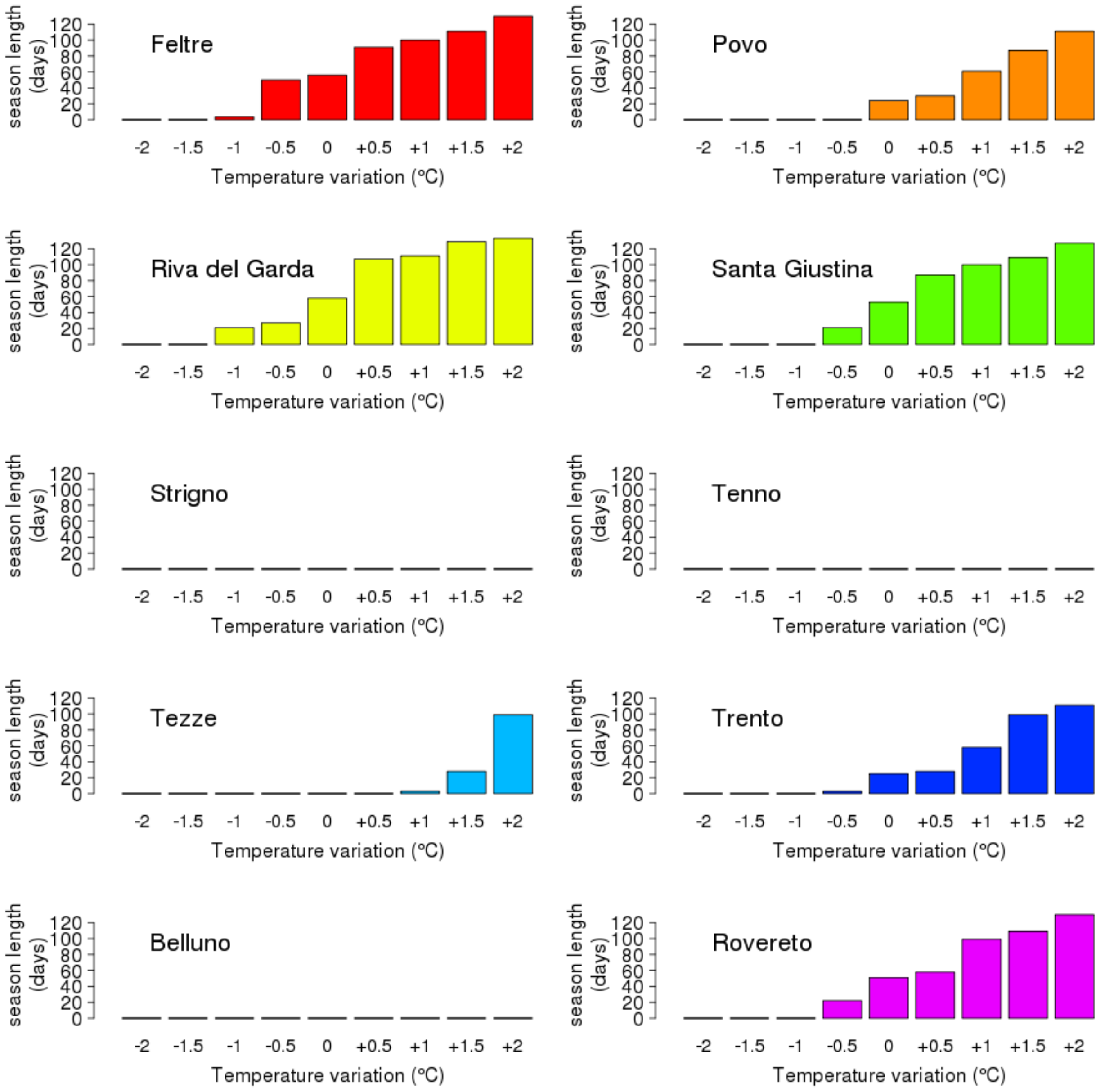

Figure S8: predicted season length for dengue with respect to changes in daily temperature

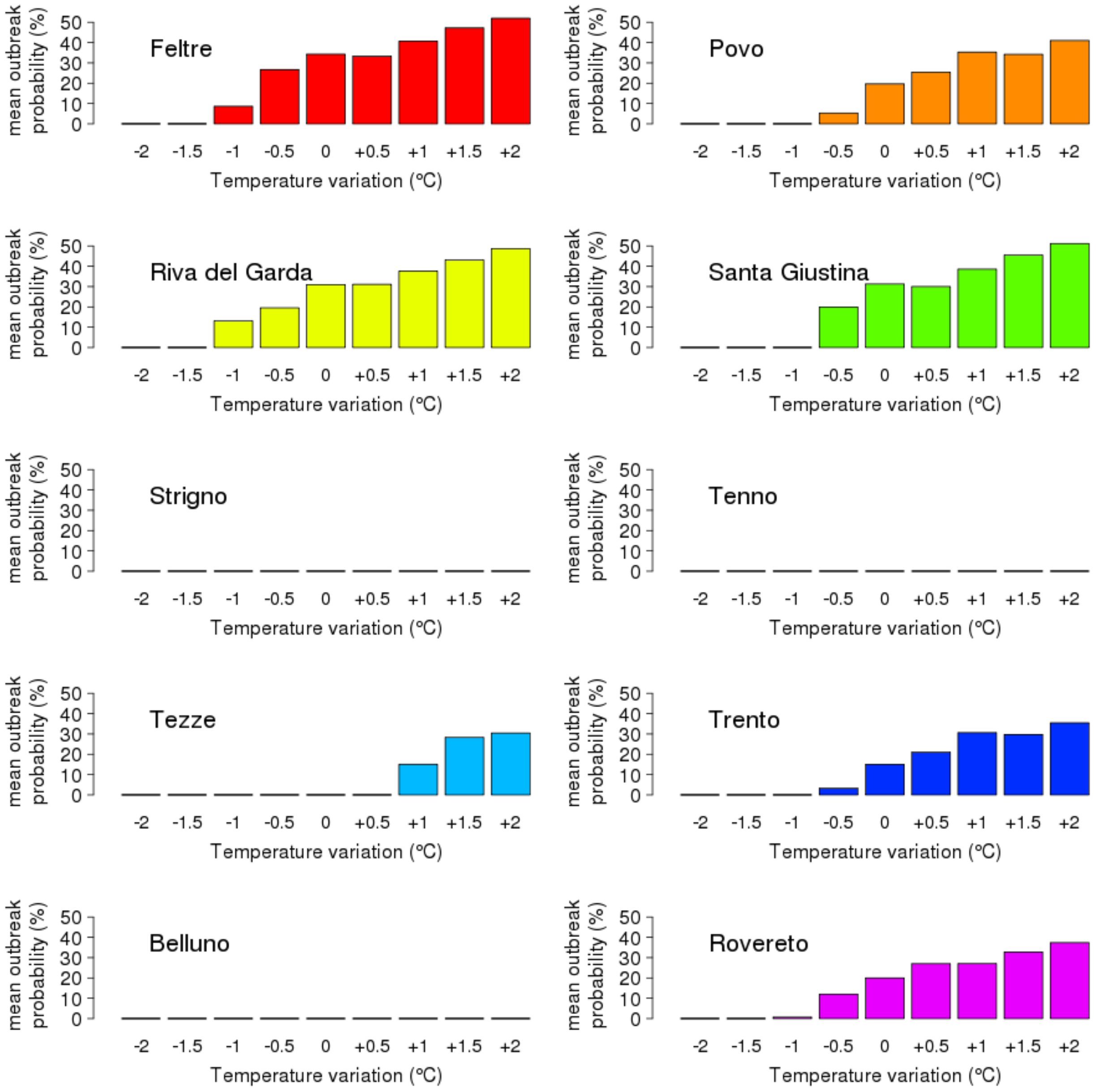

Figure S9: predicted mean probability of a dengue outbreak with respect to changes in daily temperature

In summary, model predictions of which sites are potentially at risk of a Chikungunya and dengue outbreak is robust for moderate downwards and strong upwards temperature variations; however, the risk quantification and season length can be significantly impacted by constant daily temperature changes higher than ±0.5°C.

Sensitivity analysis with respect to the biting rate

In this section, we re-compute model predictions for the outbreak probability by date of importation using different values for the biting rate. In particular, we chose as extreme scenarios the boundaries of the confidence interval reported in [2]. Figure S10 and S11 show predictions for Chikungunya and

dengue using a value of 0.05 bites per mosquito per day. In this scenario, all sites are considered virtually free from the risk of outbreaks of both infections (although the 95% confidence interval of the predicted probability may reach a peak of over 10% for Chikungunya in Feltre).

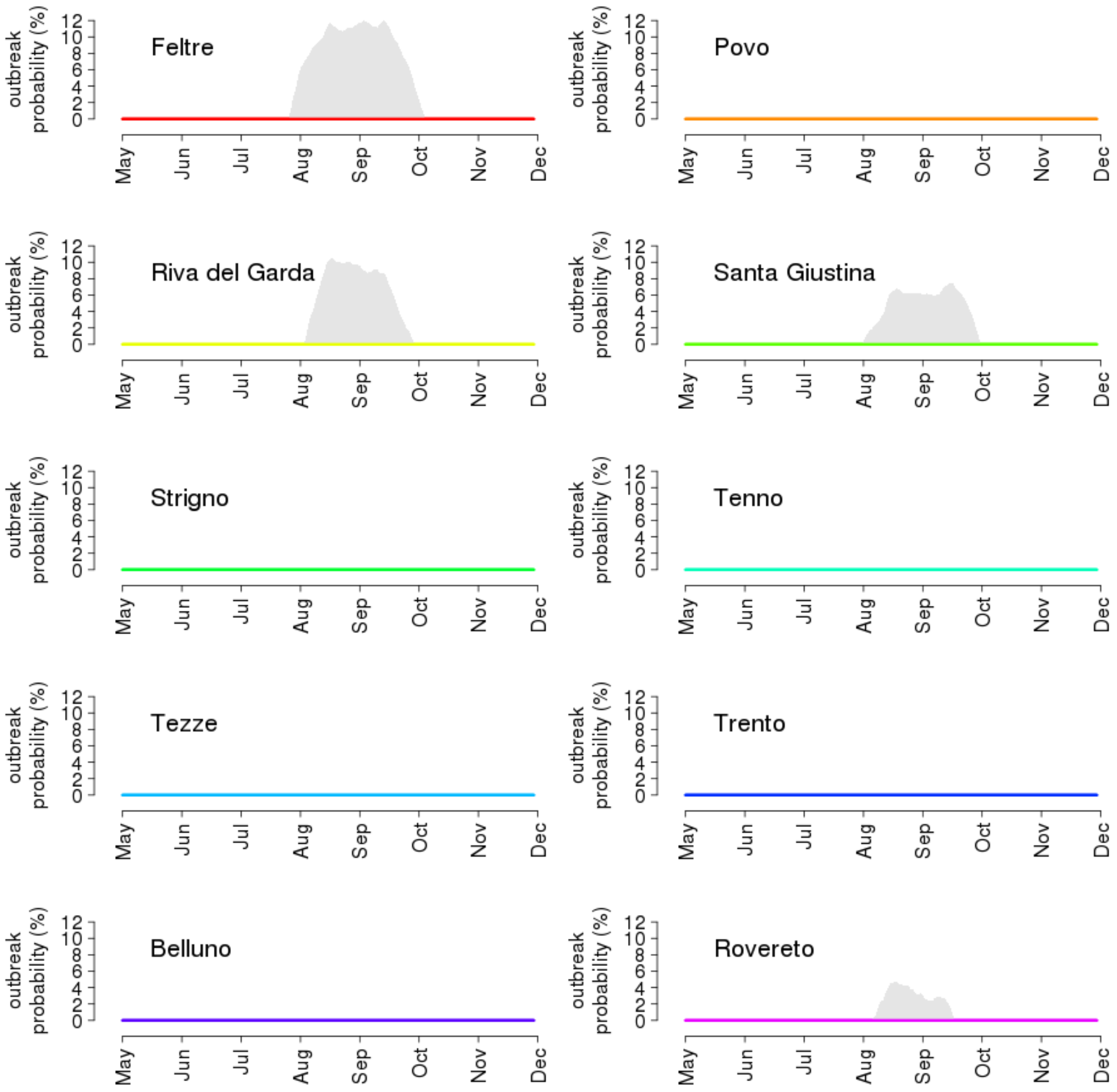

Figure S10: probability of a Chikungunya outbreak by time of first case importation for the 10 study sites, using a mosquito biting rate of 0.05 bites per mosquito per day.

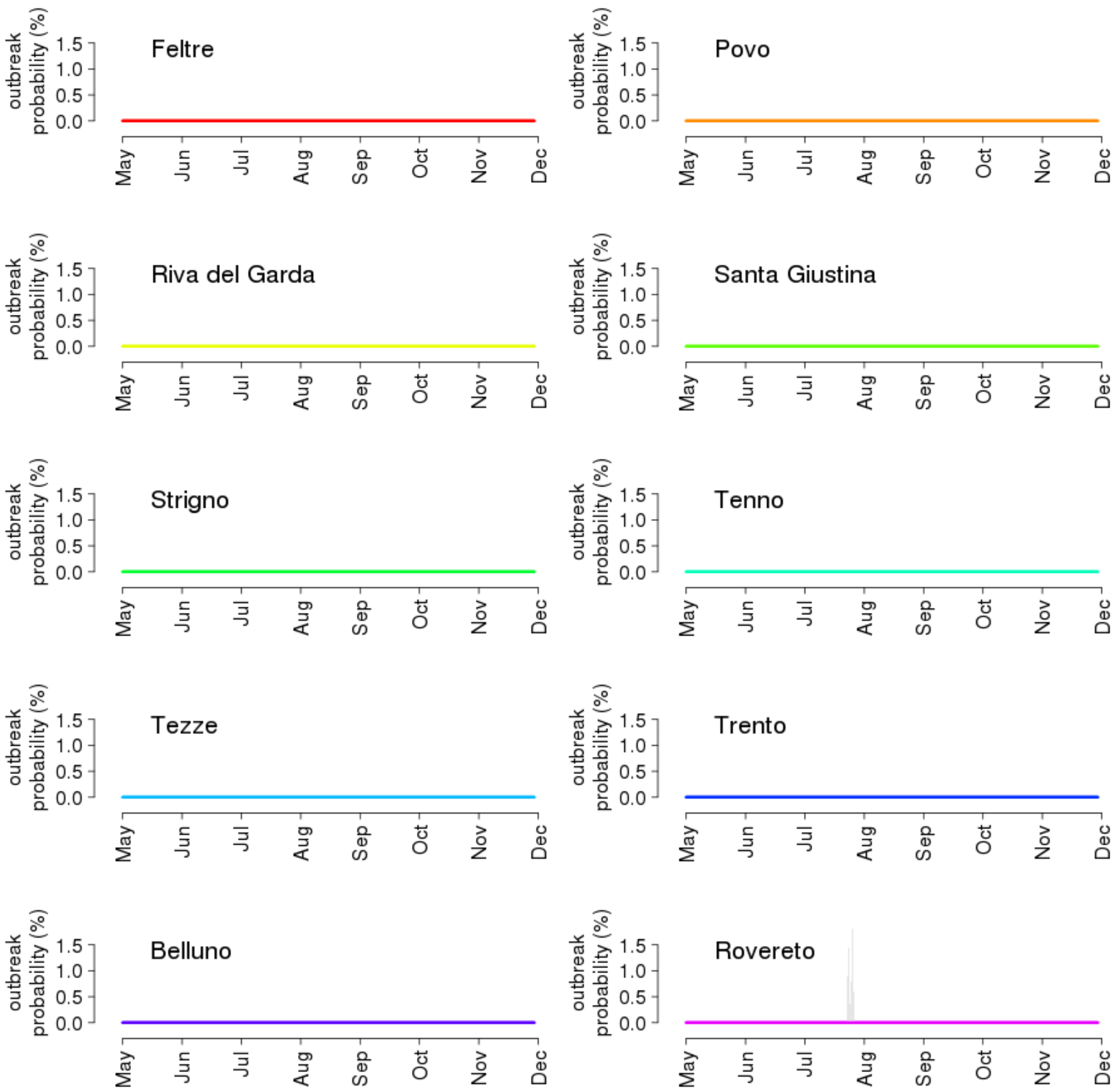

Figure S11: probability of a dengue outbreak by time of first case importation for the 10 study sites, using a mosquito biting rate of 0.05 bites per mosquito per day.

Figure S12 shows predictions for Chikungunya using a value of the mosquito biting rate 0.16 bites per mosquito per day. In this case, only Strigno has a negligible Chikungunya risk throughout the year; in several places, the outbreak probability reaches peaks as high as 60% and the Chikungunya season extends beyond the end of November.

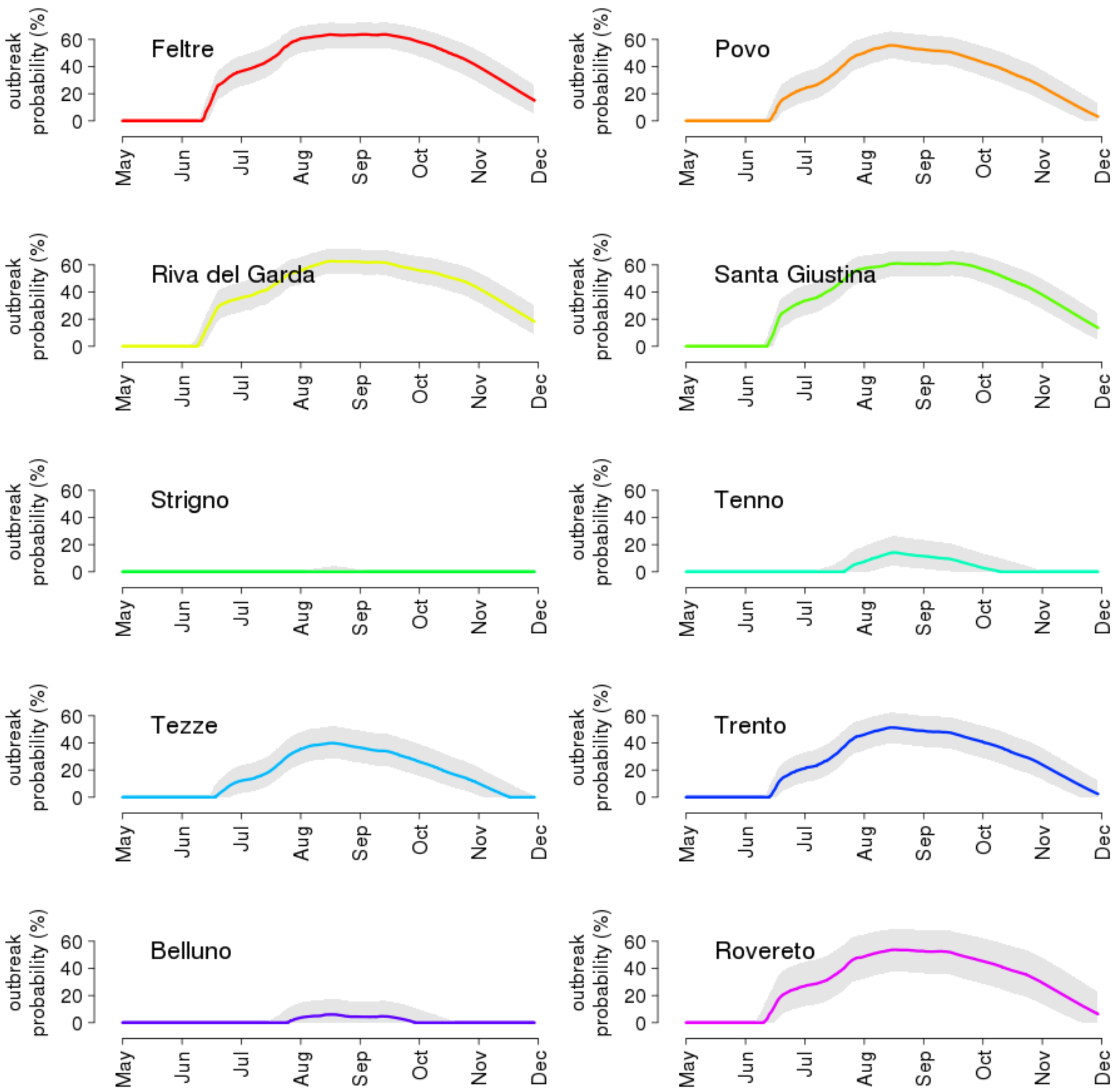

Figure S12: probability of a Chikungunya outbreak by time of first case importation for the 10 study sites, using a biting rate of 0.16 bites per mosquito per day.

Figure S13 shows the same results for the probability of a dengue outbreak under a rate of 0.16 bites per mosquito per day. In this case, peak probabilities reach values as high as 50% and the season extends from mid-June up to mid-October (as in Riva del Garda). However, three of the four sites that had a negligible risk of dengue outbreak in the main analysis (with the only exception of Tezze) conserve this status, even in this very high biting rate scenario.

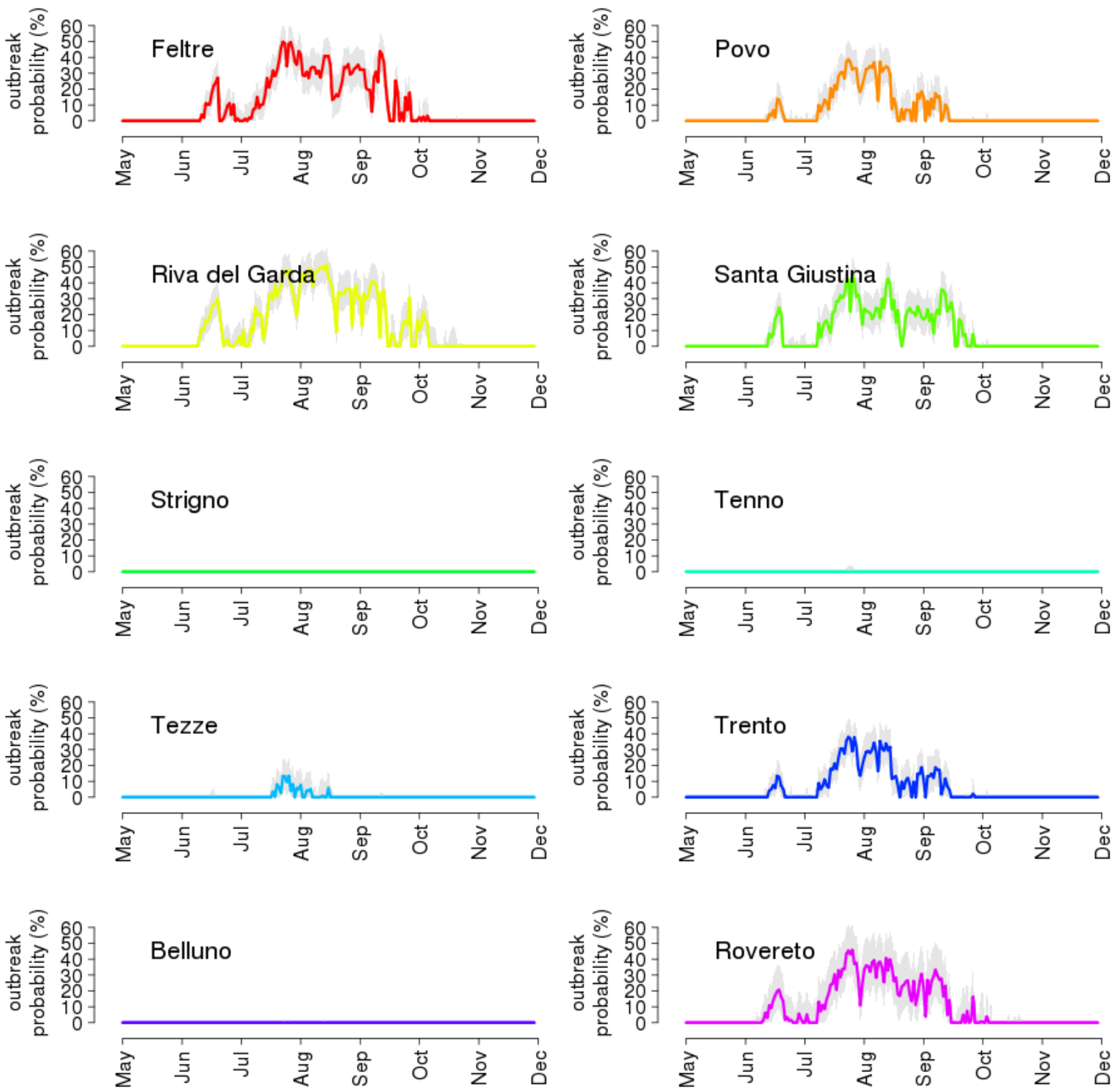

Figure S13: probability of a dengue outbreak by time of first case importation for the 10 study sites, using a biting rate of 0.16 bites per mosquito per day.

Spatialized model predictions over the provinces of Trento and Belluno

We attempted at spatializing model estimates of the mosquito abundance, using predictions from our model based on the spatial variability of temperature records in the provinces of Trento and Belluno. In the absence of a procedure for accurately spatializing the value of the larval carrying capacity (the key site-specific input parameter for the model), we built maps of the mosquito abundance by keeping its value constant over space and set to the minimum ($a = 7.7$), average ($a = 55$) and maximum ($a = 95$) values found in our 10 study sites. These three scenarios should provide a range of mosquito abundances to be expected at any site (Figures S14-S16).

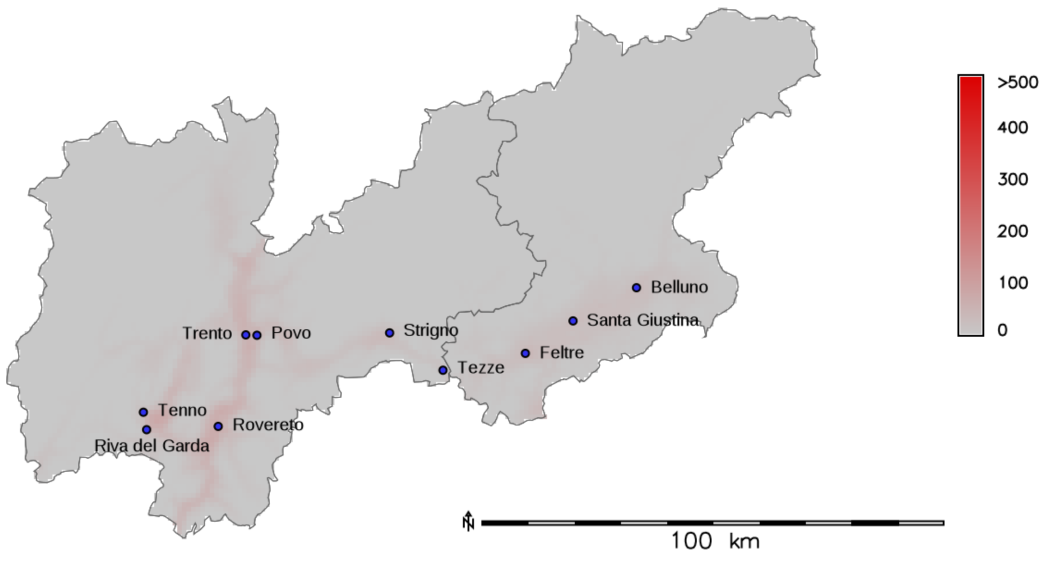

Figure S14. Predicted peak mosquito densities over the study region when using the minimum value of the larval carrying capacity (a=7.7) estimated by our model in the 10 capture sites. The maximum predicted mosquito density is 948 female adults per hectare.

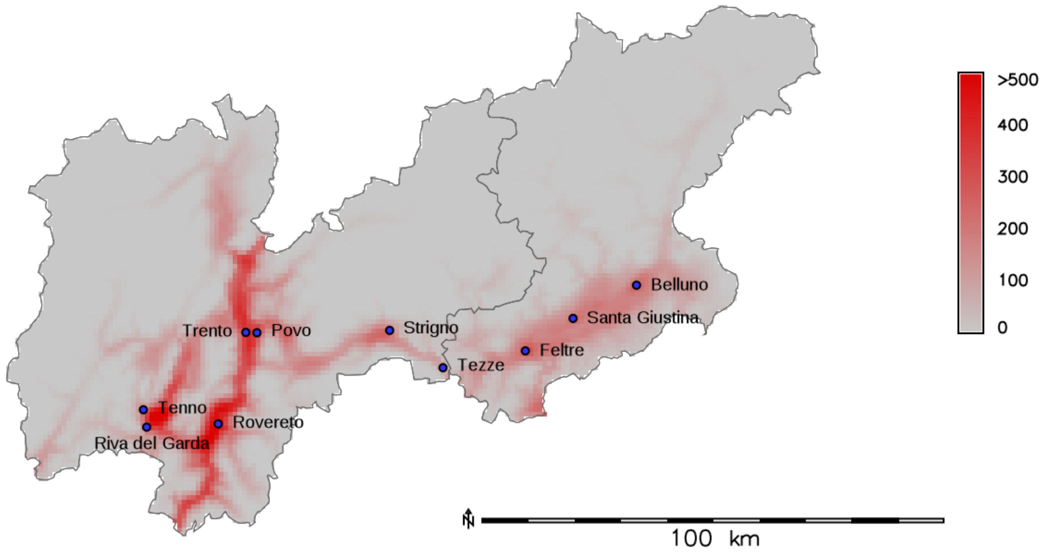

Figure S15. Predicted peak mosquito densities over the study region when using the average value of the larval carrying capacity (a=55) estimated by our model in the 10 capture sites. The maximum predicted

mosquito density is 556 female adults per hectare.

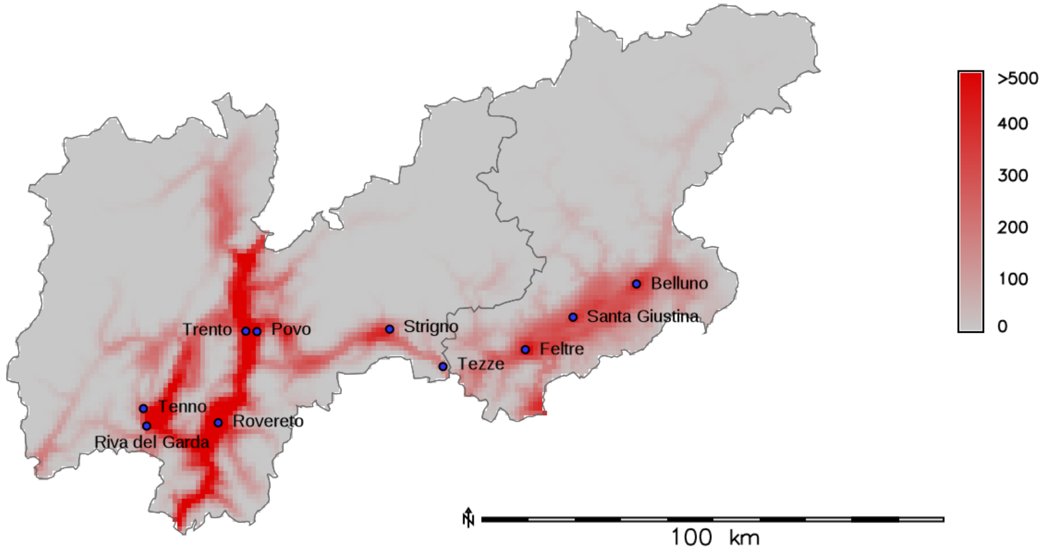

Figure S16. Predicted peak mosquito densities over the study region when using the maximum value of the larval carrying capacity (a=95) estimated by our model in the 10 capture sites. The maximum predicted mosquito density is 948 female adults per hectare.

We then built maps of the urban population density, using high-resolution information from census sections data. The Italian national institute of statistics (ISTAT) provides tables of the population density per census section and corresponding shapefiles [4]. Thus, we obtained the density of each census section by dividing its population by the corresponding shapefile area. To avoid outliers in the risk estimation, we excluded census section with population density below 10 persons / ha (representing overall less than 10% of the total population in the considered area). We then rasterized the resulting vectorial map with a resolution of 250m by computing the average of density values among census sections covered by each pixel and weighting the density by the corresponding surface contribution to the pixel.

The maps for R_0 and outbreak risks for chikungunya and dengue were then computed from the mosquito and human density maps, using the equations reported in the main text. Figures S17-S19 report peak values of R_0 for chikungunya under the different values of larval carrying capacity. Figures S20-S22 report corresponding peak for dengue.

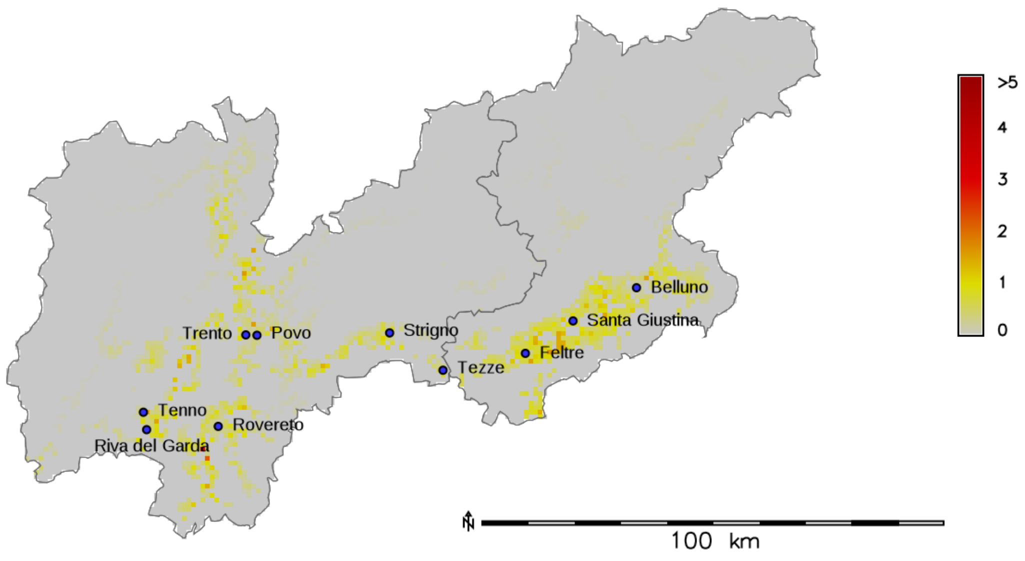

Figure S17. Predicted peak R_0 for chikungunya over the study region when using the minimum value of the larval carrying capacity (a=7.7) estimated by our model in the 10 capture sites.

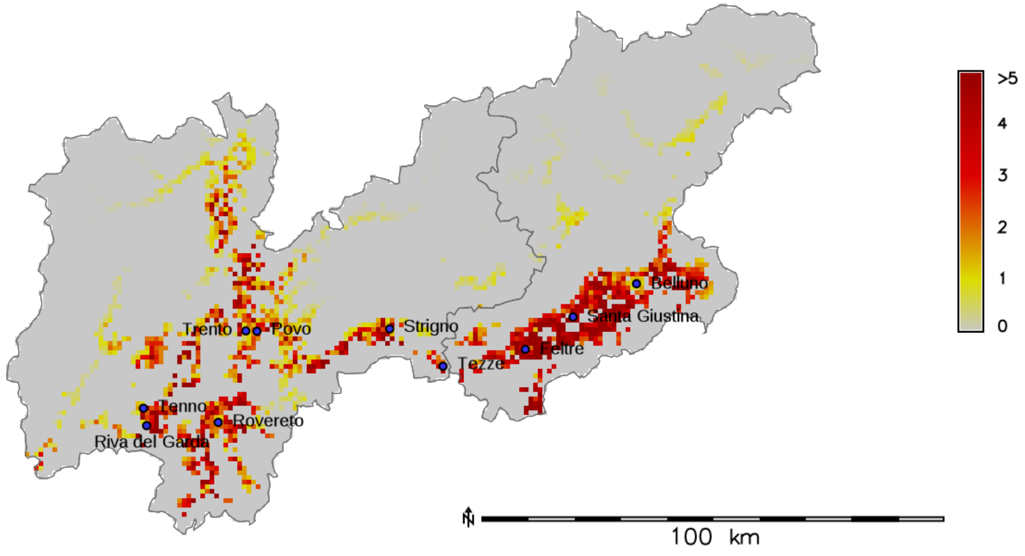

Figure S18. Predicted peak R_0 for chikungunya over the study region when using the average value of the larval carrying capacity (a=55) estimated by our model in the 10 capture sites.

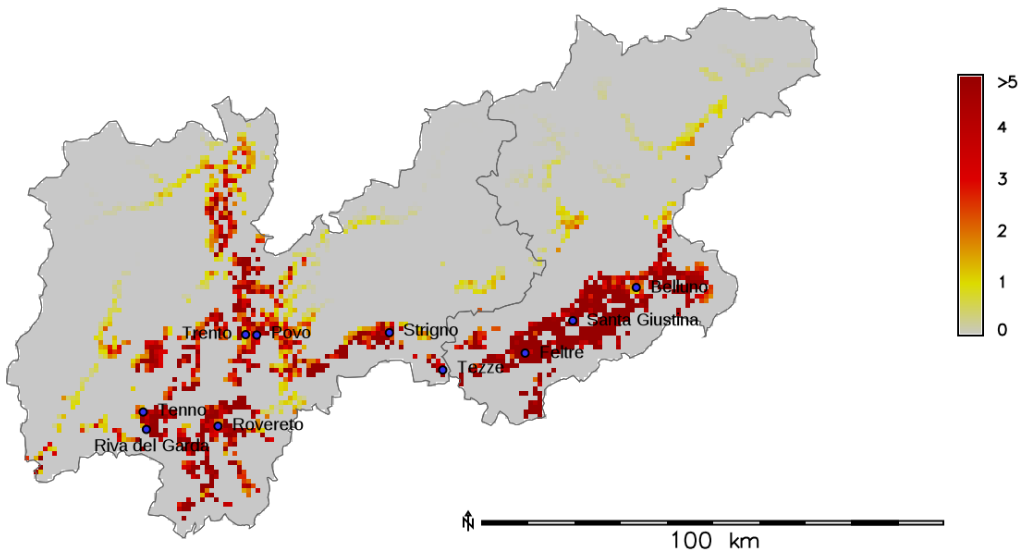

Figure S19. Predicted peak R_0 for chikungunya over the study region when using the maximum value of the larval carrying capacity (a=95) estimated by our model in the 10 capture sites.

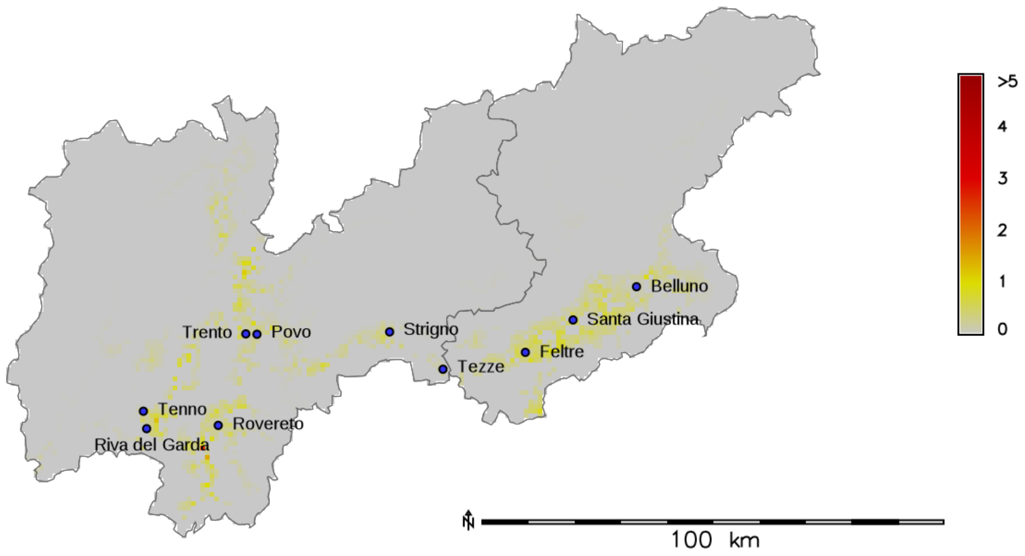

Figure S20. Predicted peak R_0 for dengue over the study region when using the minimum value of the larval carrying capacity (a=7.7) estimated by our model in the 10 capture sites.

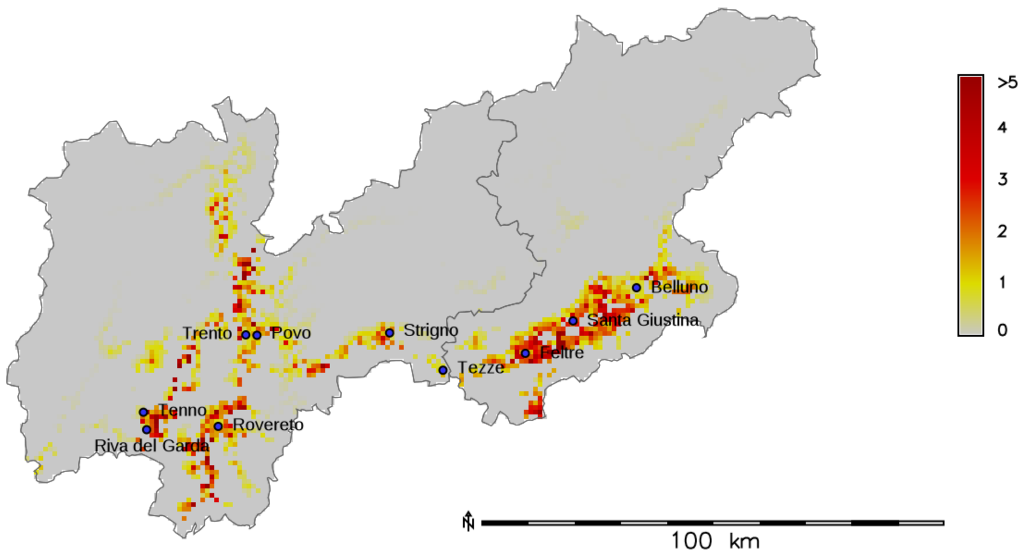

Figure S21. Predicted peak R_0 for dengue over the study region when using the average value of the larval carrying capacity (a=55) estimated by our model in the 10 capture sites.

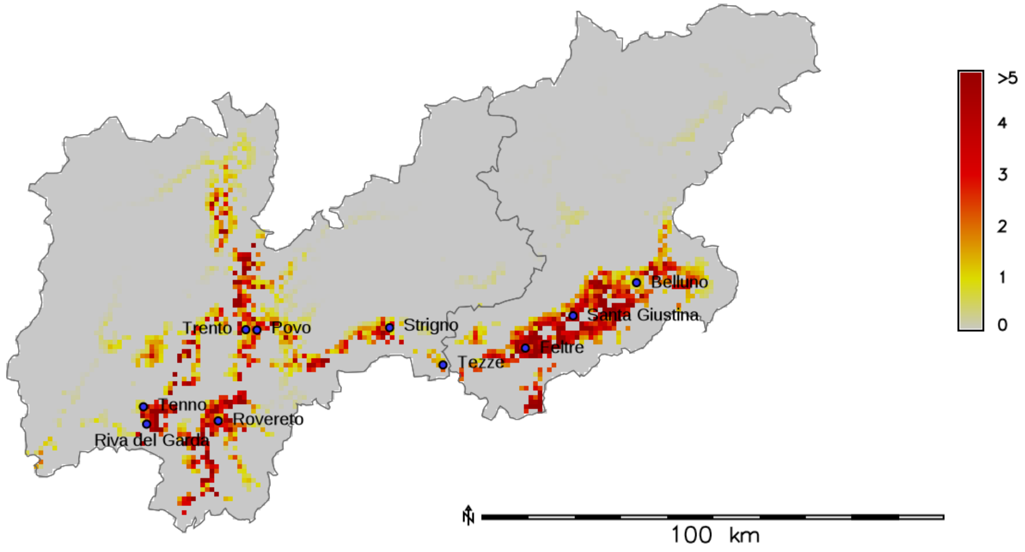

Figure S22. Predicted peak R_0 for dengue over the study region when using the maximum value of the larval carrying capacity (a=95) estimated by our model in the 10 capture sites.

Figures S23-S25 report peak values of the outbreak risk for chikungunya under the different values of the carrying capacity. Figures S26-S28 report corresponding peak values for dengue.

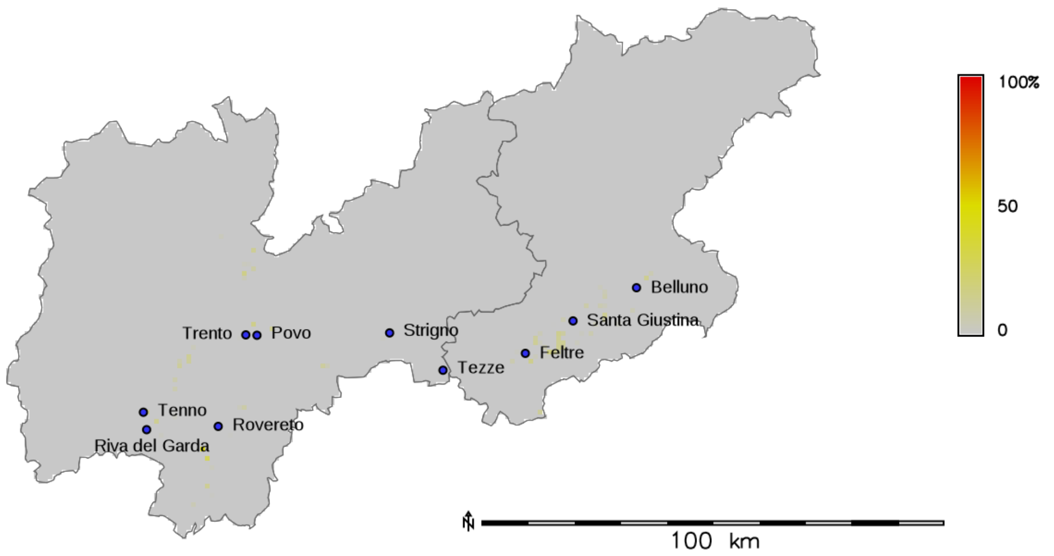

Figure S23. Predicted peak outbreak risk for chikungunya over the study region when using the minimum value of the larval carrying capacity (a=7.7) estimated by our model in the 10 capture sites.

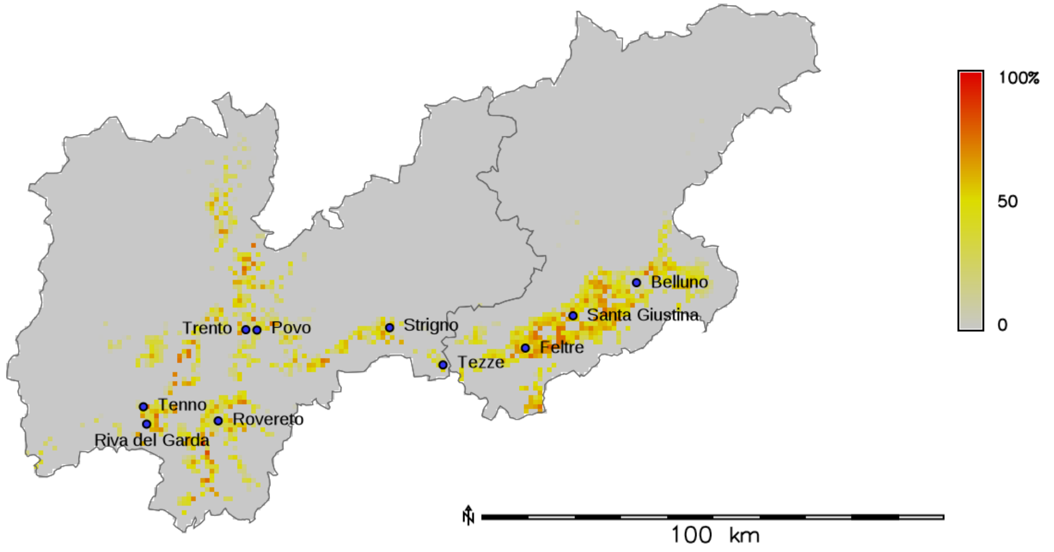

Figure S24. Predicted peak outbreak risk for chikungunya over the study region when using the average value of the larval carrying capacity (a=55) estimated by our model in the 10 capture sites.

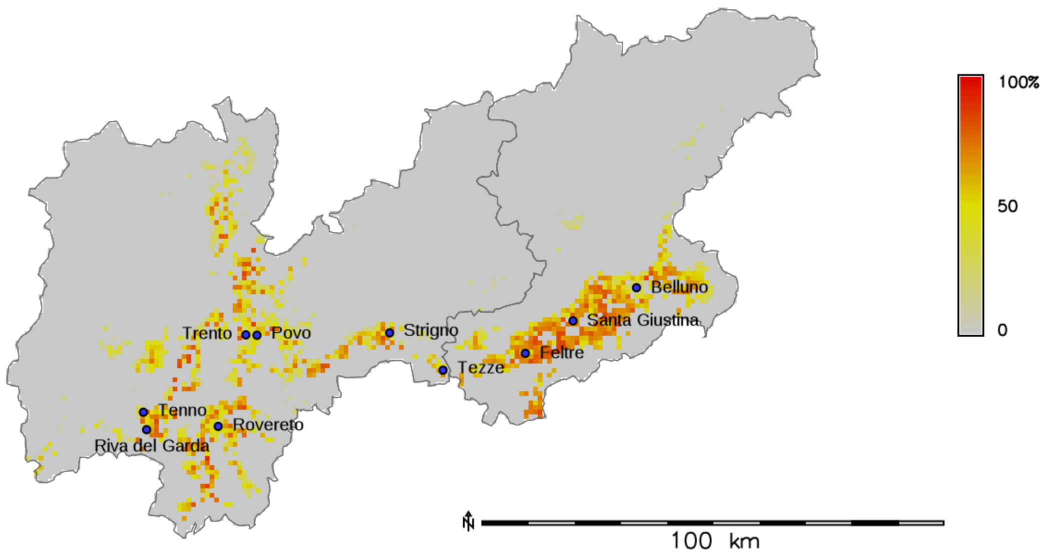

Figure S25. Predicted peak outbreak risk for chikungunya over the study region when using the maximum value of the larval carrying capacity (a=95) estimated by our model in the 10 capture sites.

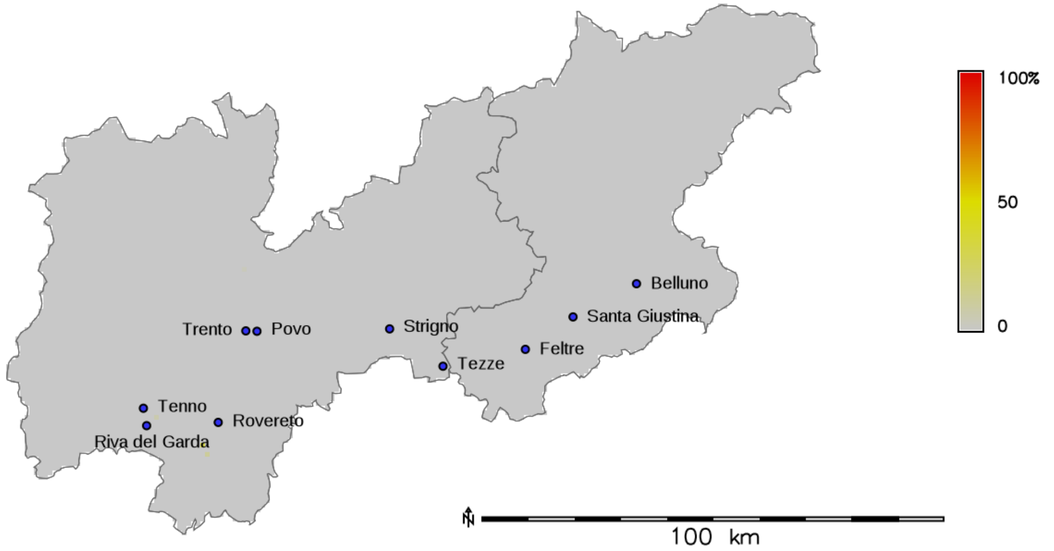

Figure S26. Predicted peak outbreak risk for dengue over the study region when using the minimum value of the larval carrying capacity (a=7.7) estimated by our model in the 10 capture sites.

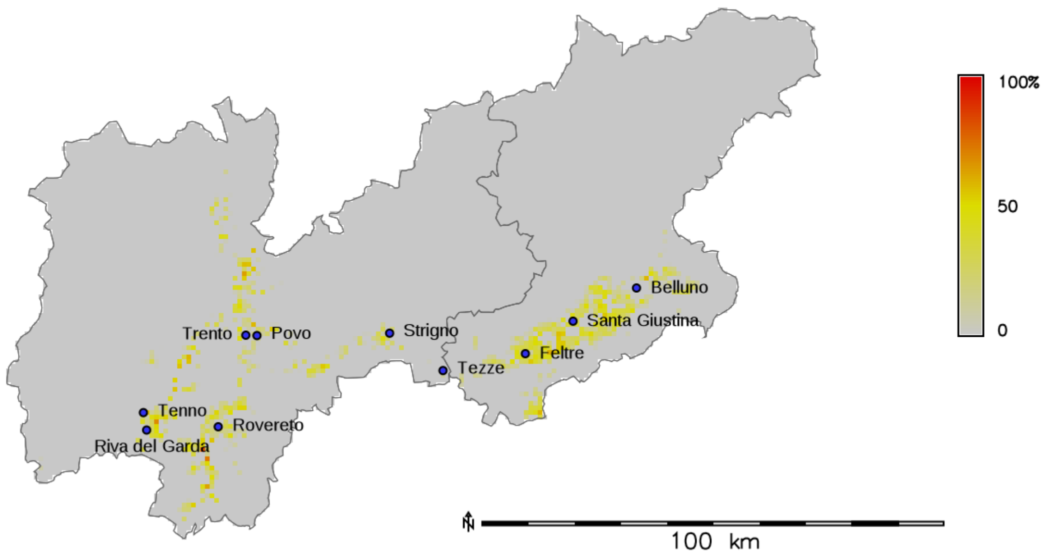

Figure S27. Predicted peak outbreak risk for dengue over the study region when using the average value of the larval carrying capacity (a=55) estimated by our model in the 10 capture sites.

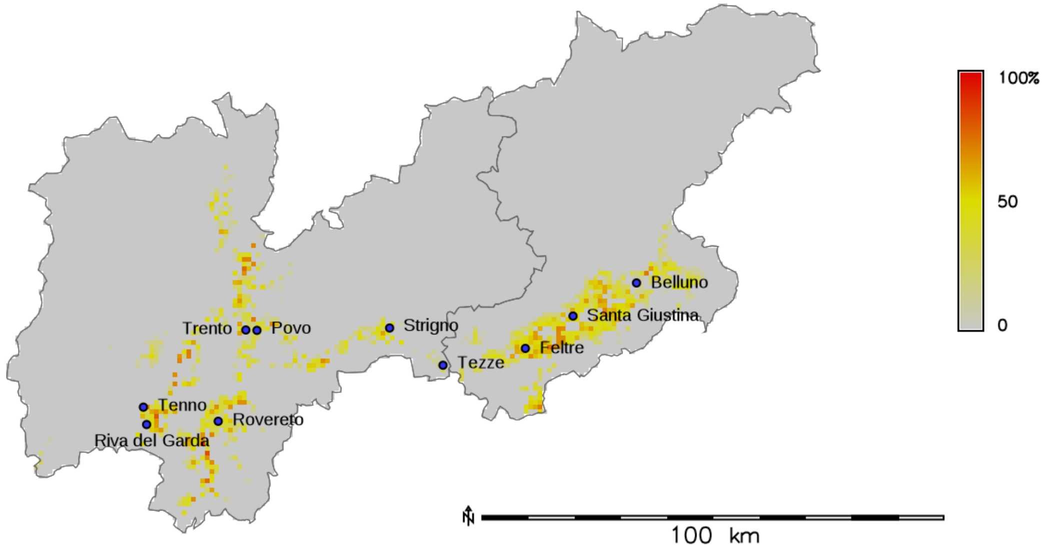

Figure S28. Predicted peak outbreak risk for dengue over the study region when using the maximum value of the larval carrying capacity (a=95) estimated by our model in the 10 capture sites.

References

1. Marini F, Caputo B, Pombi M, Tarsitani G, della Torre A. Study of Aedes albopictus dispersal in Rome, Italy, using sticky traps in mark-release-recapture experiments. *Med Vet Entomol* 2010; 24(4):361-8.
2. Poletti P, Messeri G, Ajelli M, Vallorani R, Rizzo C, Merler S. Transmission potential of chikungunya virus and control measures: the case of Italy. *PLoS One* 2011;6(5):e18860.

3. Delatte H, Gimonneau G, Triboire A, Fontenille D. Influence of temperature on immature development, survival, longevity, fecundity, and gonotrophic cycles of *Aedes albopictus*, vector of chikungunya and dengue in the Indian Ocean. *J Med Entomol* 2009 Jan;46(1):33-41.
4. ISTAT, Basi territoriali e variabili censuarie. http://www.istat.it/it/archivio/104317. Accessed April 27, 2016.
